# Prediction of potential drug targets based on simple sequence properties

**DOI:** 10.1186/1471-2105-8-353

**Published:** 2007-09-20

**Authors:** Qingliang Li, Luhua Lai

**Affiliations:** 1 Beijing National Laboratory for Molecular Sciences, State Key Laboratory of Structural Chemistry for Stable and Unstable Species, College of Chemistry and Molecular Engineering, Peking University, 100871 Beijing, China; 2 Center for Theoretical Biology, Peking University, Beijing 100871, China

## Abstract

**Background:**

During the past decades, research and development in drug discovery have attracted much attention and efforts. However, only 324 drug targets are known for clinical drugs up to now. Identifying potential drug targets is the first step in the process of modern drug discovery for developing novel therapeutic agents. Therefore, the identification and validation of new and effective drug targets are of great value for drug discovery in both academia and pharmaceutical industry. If a protein can be predicted in advance for its potential application as a drug target, the drug discovery process targeting this protein will be greatly speeded up. In the current study, based on the properties of known drug targets, we have developed a sequence-based drug target prediction method for fast identification of novel drug targets.

**Results:**

Based on simple physicochemical properties extracted from protein sequences of known drug targets, several support vector machine models have been constructed in this study. The best model can distinguish currently known drug targets from non drug targets at an accuracy of 84%. Using this model, potential protein drug targets of human origin from Swiss-Prot were predicted, some of which have already attracted much attention as potential drug targets in pharmaceutical research.

**Conclusion:**

We have developed a drug target prediction method based solely on protein sequence information without the knowledge of family/domain annotation, or the protein 3D structure. This method can be applied in novel drug target identification and validation, as well as genome scale drug target predictions.

## Background

Although great efforts have been exerted on drug research and development during the past decades, only about 500 drug targets have been identified for clinically using drugs to date[1]. Recently, this number has been revised to be 324[2], which indicates that current pharmaceutical industry actually relies on only a small pool of drug targets, compared to the large number of proteins available in human genome[3]. On the other hand, a significant number of drugs failed in the pipeline of modern drug discovery can be attributed to the wrong drug target definition at the early preclinical stages[4]. Therefore, to address new therapies by attacking novel drug targets or to predict whether a protein can be potentially used as a drug target, is extremely valuable in disease treatment, as well as the reduction of time and experimental costs in drug development.

Drug target discovery has received much attention in both academia and pharmaceutical industry. Many efforts have been made to estimate the total number of drug targets[1,2,5-8] and several drug target related databases such as TTD (therapeutic drug target database)[9], DrugBank[10], have been also established. According to the existing knowledge, classical therapeutic drug targets fell into approximately 130 protein families[2,6], which generally include enzymes, G-protein-coupled receptors, ion channels and transporters, and nuclear hormone receptors, etc[1,6]. Many groups have attempted to develop experimental and computational tools to find new potential drug targets[5,6,11-16]. Several strategies have been used in drug target prediction, which can be generally divided into two groups. The first group is to analyze the known therapeutic drug targets from genome level based on sequence homology or domain containing method [5,6], which takes protein families into account to find potential novel drug target family members. In fact, not all proteins in the same family can be used as drug targets. The other one is to search for binding pockets on the protein surface based on protein 3D structures, and to identify those that may bind to drug-like compounds with reasonable affinities[11,13]. Technically, this kind of methods is limited to the availability of 3D structures and cannot be applied to genome scale. Recently, Han et al. [16] used machine learning methods to build a model with 1,484 clinical and research drug targets from TTD database[9], and predicted druggable proteins among different organisms.

Clearly, the quality of drug target data restricts the predictive power of models. Unfortunately, several versions of drug target lists have been proposed[1,2,5-8]. Therefore, we have to establish a critical criterion to select valid drug targets for the prediction. The possible reasons for many versions of drug targets are: the definition of drug target is difficult and also arbitrary[7]; it is difficult to assign each drug to its target due to poorly understood pharmacology, limited selectivity against related proteins and some targets are even multimeric protein complex where the same subunits can unite in different combinations to form different targets[2,5]. In this study, we follow the definition of drug target by Imming et al[7], "...a target to be a molecular structure (chemically definable by at least a molecular mass) that will undergo a specific interaction with chemicals that we call drugs because they are administered to treat or diagnose a disease. The interaction has a connection with the clinical effect(s)." The version of drug targets used in this study has been collected by Overington et al[2], who propose a consensus version of 324 drug target from a comprehensive survey on earlier reports. Considering the differences in mechanisms between human origin drug targets and pathogen origin targets, and the differences of amino acids composition among different organisms, only human origin drug targets are used in the current study. As the main purpose of the present study is to predict potential drug targets for drug design and screen of chemical compounds, consequently, only 186 targets of FDA-approved oral small-molecular drugs are taken into account. Using simple physicochemical properties extracted from protein sequences, several support vector machine (SVM) models have been constructed with critical testing. The best SVM model has been applied to large scale datasets, and some of the predicted potential drug targets have attracted much attention in experimental studies.

## Results and discussion

### Kernel function selection

Three commonly used kernels, linear, polynomial and radial basis function (RBF) were tested in order to find the best performance SVM model. 10-fold cross-validation was done to evaluate the performance of each kernel functions. A 146-dimension vector of physicochemical features (in Table 1) was used to represent protein sequence. The results of the three kernel functions with training set (1) are listed in Table 2.

**Table 1:** Protein sequence based features used

Dimension	Properties
20	Composition of the 20 amino acid residues
21	Hydrophobicity
21	Polarity
21	Polarizability
21	Charge
21	Solvent accessibility
21	Normalized *van der Waals *volume

**Table 2:** 10-fold cross-validation results^a ^of the SVM with different kernel functions

Kernel function	Sensitivity	Specificity	Accuracy
SVM-Linear	84.03 ± 0.74	74.90 ± 1.47	79.46 ± 0.87
SVM-Polynomial	84.56 ± 1.06	76.38 ± 2.14	80.47 ± 0.87
SVM-RBF	85.91 ± 2.60	80.54 ± 2.64	83.22 ± 1.76

^a ^SVMs with different kernel functions were trained with training set (1).

The best performance of the three kernel functions is about 80% in overall accuracy. The sensitivity (percentage of recognized positive samples among all positive samples) is around 85%, which is 5–9 percent higher than the specificity (percentage of recognized negative samples among all negative samples, from 75% to 80%). The performance of the SVM with linear kernel was a little worse than that of polynomial kernel. SVM with RBF kernel outperformed the other two, which gave an overall accuracy of 83%, sensitivity of 85% and specificity of 80%, respectively.

Systematic searching of the parameter space was carried out for each of the three kernels in model optimization. For linear kernel, only one parameter, the error tradeoff C, was tuned; for polynomial kernel,

*k*(*x*,*x'*) = ((*x *• *x'*)*s *+ 1)^*d*^

the two kernel parameters and error tradeoff C were tuned, and the parameter *d *was fixed at a value of 5 as in the previous studies[17,18]; for RBF kernel,

*k*(*x*,*x'*) = exp(-*v*||*x*-*x*'||^2^)

parameter ν and error tradeoff C were optimized.

From the results shown in Table 2, only the SVM model with RBF kernel function was chosen for the subsequent computations in this study.

### Training set selection

Although good results were achieved for training set (1), the specificity was relative low. Therefore, one question might be raised – "are 186 samples enough to cover the entire non drug-target space?" Considering the diversity of the putative non drug-target proteins, the non drug-target space might not have been sampled completely. Therefore, we have constructed five additional training sets, training set (2) – (6), in which the ratios of the number of positive samples to negative ones became 1:2, 1:4, 1:6, 1:8, 1:10 instead of 1:1 in the training set (1). The same strategy used in training set (1) was applied to these five training sets.

**Figure 1:**
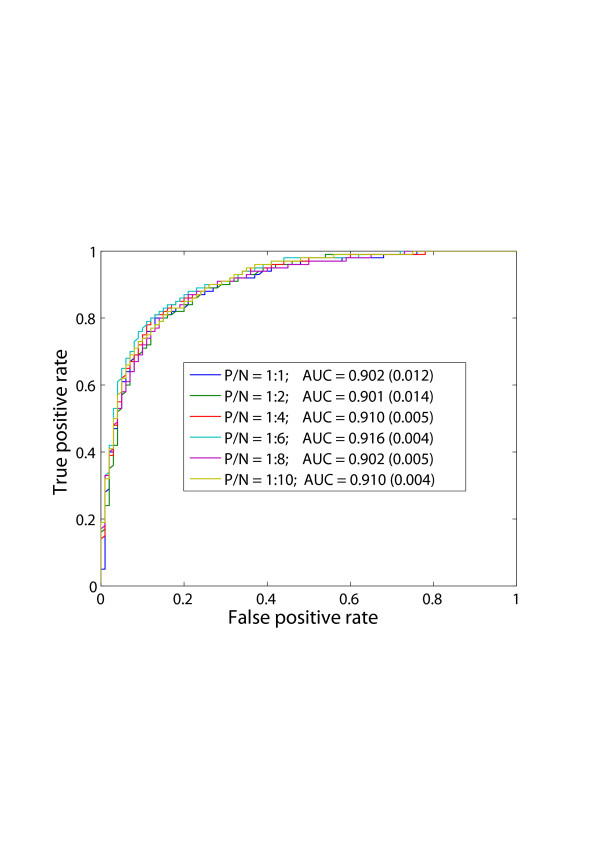
**ROC curves of training set (1) – (6) by RBF-SVM using 10-fold cross-validation**. P and N indicate positive and negative, respectively. AUC means the area under the curve. The larger the AUC is, the better the performance of the model will be. The real numbers in brackets are standard deviation (std).

The 10-fold cross-validation results of all six training sets are listed Table 3 and the relevant ROC curves are shown in Figure 1. The six ROC curves are almost overlapping in Figure 1, and the area under the curve (AUC) varies between 0.900 and 0.920, which demonstrates that the performance of the SVM model does not change significantly with the increase of negative samples in the training set.

**Table 3:** 10-fold cross validation results with different training sets

Training set	Positive/Negative	Sensitivity	Specificity	Accuracy
1	1:1	85.91 ± 2.60	80.54 ± 2.64	83.22 ± 1.76
2	1:2	82.28 ± 1.02	82.82 ± 2.67	82.64 ± 1.66
3	1:4	81.88 ± 1.34	84.17 ± 1.78	83.71 ± 1.48
4	1:6	81.61 ± 0.77	84.73 ± 2.38	84.28 ± 2.10
5	1:8	82.01 ± 1.64	84.57 ± 1.68	84.29 ± 1.64
6	1:10	82.69 ± 0.74	83.72 ± 1.20	83.62 ± 1.14

**Figure 2:**
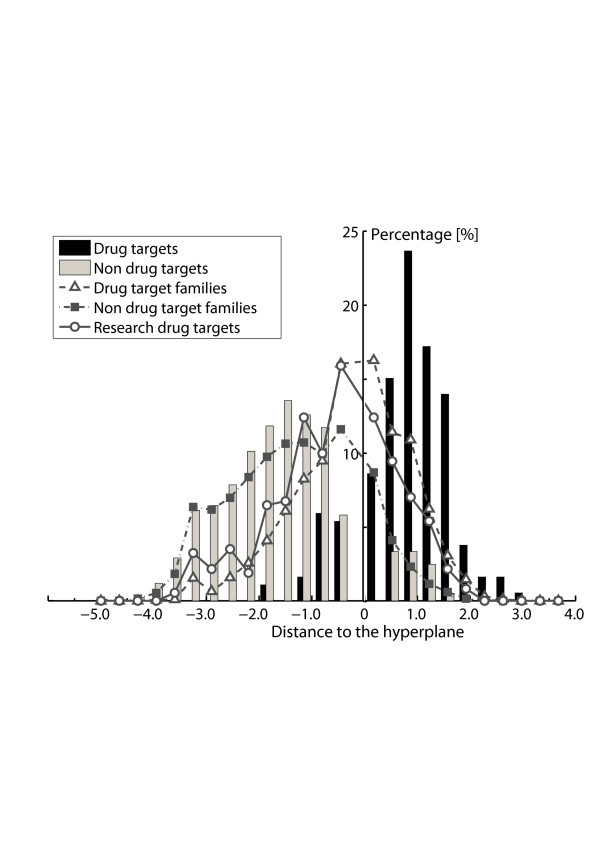
**Distribution of predicted distance to the hyperplane**. The distance to the hyperplane of a support vector machines model is related to the confidence of the predicted results. The bigger the distance is, the more confident the predicted results will be. The histogram is the prediction results for the drug targets and non drug targets in the 10-fold cross-validation of training set (4) – (positive/negative = 1:6). Drug target families contain 3,444 human original proteins from Swiss-Prot, which are in the same family of known drug targets. Non drug-target families consist of 9,758 putative non drug targets. Research drug targets contain 371 human origin research drug target proteins which do not belong to the known drug target family.

Despite of the similarities, we can still observe several changes due to better sampling of the non drug-target space. The area under the curve (AUC) increases from about 0.90 to 0.91 and remains around 0.910 along with the increase of negative samples. At the same time, the standard deviation of under the curve (AUC) is getting smaller and seems to converge to about 0.005. Similar trend can be observed from Table 3, where the specificity increases from 80% to 84% and the sensitivity drops down from 85% to about 82%. Starting from training set (3) (Positive/Negative = 1:4), the performances of SVM models become stable. The reason why the sensitivity drops down is because that the training sets become unbalanced (positive/negative = 1:1 is called balance) along with the increase of the number of negative samples, in that way the SVMs tend to push the hyperplane towards the side with a small number of samples[16] (refer to histogram in Figure 2). It should be noted when the training set is unbalanced, if the sensitivity and specificity are not close to each other, then the overall accuracy will not suitable for evaluating model performance. For example, if positive/negative = 1:9, even all testing samples are predicted to be negative, the overall accuracy will still be 90%, which actually is not the case.

Considering the analysis above, training set (4) (positive/negative = 1:6) was chosen for the following calculations. The confidence of a support vector machine prediction is related to the distance to the hyperplane in SVM model. Larger distance equates to higher confidence. Figure 2 illustrates how this model performed in 10-fold cross-validation. For drug targets, the method is about 82% accurate; for putative non drug targets, the accuracy is about 85%.

### Feature selection

Generally speaking, choosing a more relevant subset of features leads to a simpler model, which can often lead to better performance in machine learning[16,19]. This might come from the noisy features that make fitting the hyperplane more complex. Here, we used F-score, a simple and intuitive method to measure the discrimination of each feature implemented as a tool in Libsvm package[20]. The larger F-score is, the more discriminative the feature is.

**Figure 3:**
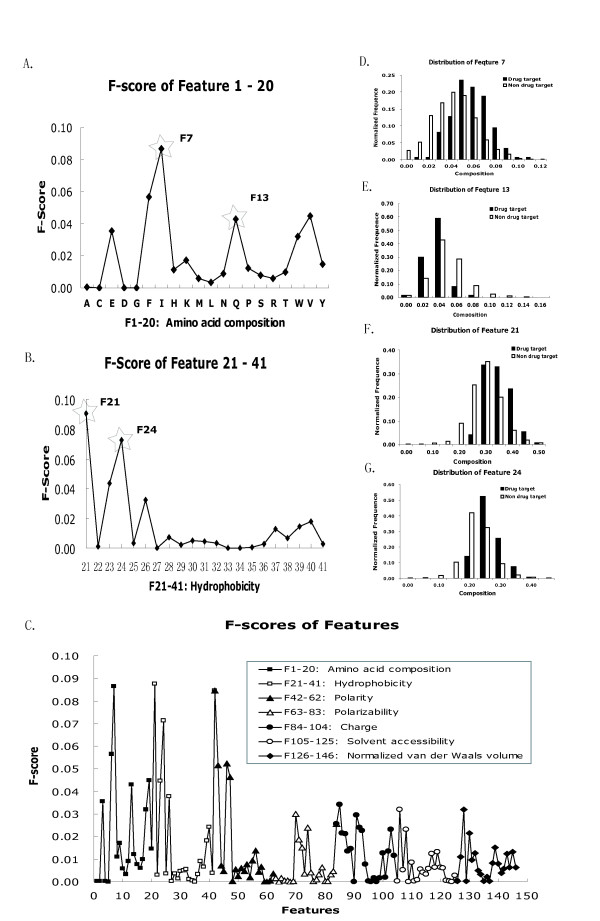
**The F-scores of features and relevant distribution**. A. F-scores of the first 20 dimensional features that are the standard amino acid composition. The distribution of the 7th and 13th feature with star marked, F7 and F13 which are the composition of amino acid of Ile and Gln, is illustrated in D and E. B. F-scores of the next 21 dimensional features that are the hydrophobicity of protein sequence. The first three dimensions, F21-23, represent compositions of hydrophobic, neutral and polar amino acid, respectively; F24-26, represent the percentage of transition frequency between hydrophobic, neutral and polar amino acid residues; F27-41 are which the first, 25%, 50%, 75% and 100% of the amino acids of a particular group are located. The distribution of feature F21 and F24 with high F-score are shown in F and G. C. The F-scores of complete 146 features.

The F-scores of each feature are shown in Figure 3C and several features with higher F-scores are also illustrated in Figure 3D–G. It can be seen that hydrophobicity and polarity are the most discriminative features. The hydrophobic residues Phe, Ile, Trp and Val, together with polar residues, Glu and Gln tend to have higher F-scores than other residues in Figure 3A. Similarly, high F-scores of F21, F42 and F43 can be observed, which are related to hydrophobic and polar group amino acids. In addition, the percentages of transition frequencies between hydrophobic amino acids and neutral amino acids, polar amino acids and neutral ones also seem to be discriminative features. One of the reasons why such features are more discriminative might be due to the large percentage of transmembrane proteins in known drug targets, which have more hydrophobic trans-membrane regions.

With careful feature selection, we observed that there were no obvious improvements (less than 1%) of the SVM model. Although there was a certain correlation between some features, this level of correlation would not substantially reduce the model performance in this study. Similar results were obtained in previous study[16], therefore, all the 146 features were selected. It should be emphasized that despite of many advantages of F-score method, it dose not reveal the mutual information among features. As contrast, RBF kernel SVM can non-linearly combine each of the features and achieved good results in the present study.

Furthermore, one might wonder the performance of the model if only 20 features of amino acid composition are taken into account. This was tested on training set (4) (Table 4). Both sensitivity and specificity are around 80%, which are only 2% and 4% lower than those of 146-feature model, respectively.

**Table 4:** 10-fold cross-validation results^a ^with 20 features of amino acid composition

Training set	Positive/Negative	Sensitivity	Specificity	Accuracy
4	1:6	79.20 ± 2.47	80.78 ± 1.84	80.56 ± 1.90

^a ^It is built on training set (4) – positive/negative = 1:6

### Further evaluation of model with blind test

Blind test was employed to further evaluate the performance of the model generated above. The testing dataset consisted of 37 drug target proteins (positive) and 223 putative non drug-target proteins (negative). The model successfully recognized 31 drug targets among all the 37 drug-target proteins and 188 of the 223 putative non drug-target proteins on average (in Table 5). The sensitivity and specificity are about 84% and 85%, respectively, which are very close to the results obtained from the 10-fold cross-validation test. The consistency of the performance in both cross-validation test and blind test, demonstrates the validity of the present method. Therefore, we conclude that this model has good predictive power in critical testing process.

**Table 5:** Blind testing results^a^

Positive	Negative	Sensitivity	Specificity	Accuracy
		83.79 ± 6.24	84.55 ± 2.60
37	223			84.60 ± 1.84
		(31 ± 2)/37	(188 ± 6)/223	

^a ^The model is trained with training set (4) (positive/negative = 1:6) using RBF kernel function.

**Figure 4:**
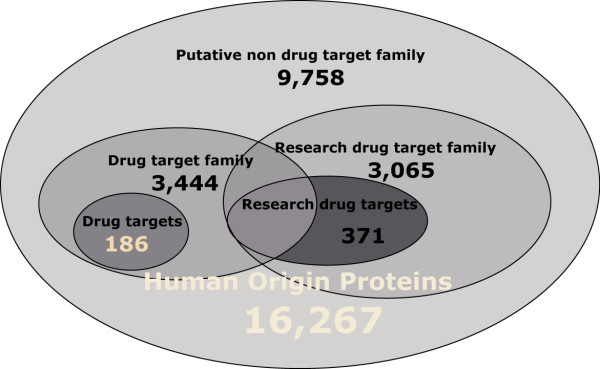
**The relationship of different datasets**. 16,267 human origin proteins are from Swiss-Prot. 186 drug targets are the targets of FDA-approved oral small-molecule drugs of human. The other four datasets are 3,444 drug target family members, 371 research drug targets with the ones in drug target family excluded, 3,065 proteins from research drug target family and 9,758 putative non drug targets, which are predicted respectively.

Since the current method provides pretty good performance in the critical testing of the 10-fold cross-validation test and blind test, one might wonder how it performs in larger dataset and whether it can be used in novel drug target prediction at genome scale. In order to answer these questions, another four datasets were prepared, dataset I – IV, which contained proteins of drug-target family members, research drug targets, research drug-target family members, and putative non drug targets, respectively. The relationship of these datasets is shown in Figure 4.

### Prediction results on dataset I – drug target family

This dataset contains 3,444 proteins from 142 drug-target families from Pfam annotation[21], among which 1,557 proteins were predicted as potential drug targets. The percentage of predicted potential drug targets was about 45% and more than half of the proteins were recognized as non drug targets. The distribution of the predicted distance to the hyperplane is illustrated (blank triangle in broken line) in Figure 2. Interestingly, 60% of the samples distribute in the range of -1.0 to 1.0, which means a large potion of the proteins in the drug target family are on the margin between drug targets and non-drug targets. In other words, drug target family dataset seems closer to drug target dataset than putative non drug-target family dataset does.

### Prediction results on dataset II – Research drug target

371 human origin research drug targets selected from TTD[9] and DrugBank[10] were included in this dataset, while the other research drug targets which belong to drug target families were excluded. 140 research drug targets out of 371 were predicted as drug targets and the percentage is about 38%. The distribution of the predicted distance is also shown in Figure 2. The distribution looks like that of the drug target family to some extent. Similarly, research target dataset seems more like drug target dataset than the putative non-drug target dataset does. But, it looks less like drug target dataset compared to drug target family data due to a slight shift of the distribution curve to the negative direction.

### Prediction results on dataset III – Research drug target family

Similarly, the research drug target family proteins were also predicted. This dataset consists of 3,065 proteins. 797 proteins out of 3,065 research drug target family members were predicted as drug targets and the fraction is 26%, which is slight higher than that of the putative non drug targets (15%, in the next subsection). The dataset looks more like non-drug target dataset than the other two do.

### Prediction results on dataset IV – Putative non drug target

This dataset contained 9,758 putative non drug-target proteins. 8,375 proteins out of the putative non drug targets were successfully recognized as non drug targets with the accuracy of about 85%. The distribution of the predicted distance is also shown in Figure 2 (filled square in dot line). The distribution is very similar to that of the putative non drug target in the training set. This indicates that the sampling by the non drug-target proteins in the training set (training set (4)) completely covered the entire putative non drug-target dataset to some extent. This may also answer the question why the performance remains constant when increasing the negative samples of the training set.

It should be noted that the "putative" non drug-target dataset does not mean that proteins in this dataset are actually non drug targets, and it only shows they are not drug targets according to the existing knowledge. Thus, one might not expect a perfect classification of this dataset. As a matter of fact, to identify novel drug target (or target family) is the main focus in this field, and it is also one of the main purposes of this study. Therefore, it is worthy to check the misclassified part of this dataset, as the commonly used homology based or domain based annotation method can not detect this part of potential drug targets.

**Figure 5:**
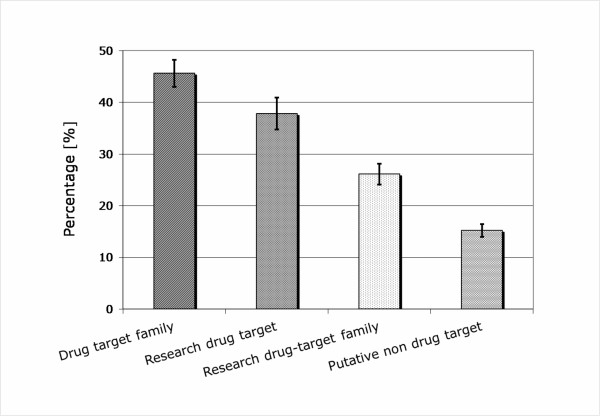
Prediction results on four larger datasets.

The results of the predicted drug targets on dataset I – IV are illustrated in Figure 5. Drug target family is well known resource for discovery of novel potential drug targets[4], which is also supported by the current study. 45% proteins of this dataset were predicted as potential drug targets, which is the highest of the four and almost two times higher than that of research target family. On the other hand, this shows that not all proteins from drug target family can be used as drug target, in fact more than half cannot according to the present study. Although the research drug target family dataset was predicted to contain only 26% potential drug targets, the research drug target dataset was predicted to contain about 38% potential drug targets, indicating that the research drug targets are the well chosen ones from the research drug target families that were put into experiment studies.

As to the putative non drug target dataset, 15% of the proteins were predicted to be drug targets. This part of 'misclassified' proteins are of great value as they might become potential novel drug targets or provide import information in drug target identification and validation research. The top 30 predicted potential drug targets with the distance to the hyperlane higher than 1.7 are listed in Table 6 (the other predicted potential drug targets are listed in Additional file 1). Some of them have been reported as potential drug targets in the literatures. For example, No.1, 3, 6, 16, 24 are five Frizzled receptors (Frizzled-2, 3, 4, 7, 9), belonging to G-protein coupled receptor Fz/Smo family, which are the receptors for Wnt protein[22]. Wnt signalling has been reported to be related to various diseases, such as kidney damage, leukaemia, metastasis, schizophrenia, cancer and so on, where Frizzled receptors are considered as one of the promising therapeutic targets [23-26]. No.4 is Smoothened homolog (SMO), also belonging to G-protein coupled receptor Fz/Smo family, which is involved in the Hedgehog signalling, a pathway leading to pathological consequence in various human tumours, such as gastric cancer and pancreatic cancer[27]. It has also been reported that cyclopamine as SMO inhibitor is a potential "mechanism-based" therapeutic agent for the treatment of these tumours[27,28]. No.7, NADPH oxidase 3 (NOX3), is almost exclusively expressed in the inner ear[29], which is a potential drug target in ROS (reactive oxygen species) related hearing loss[30]. In addition, No.21, P2X purinoceptor 4 (P2X4), has recently been implicated in pain sensation, which is considered as a new target to treat neuropathic pain in current research[31].

**Table 6:** Predicted drug targets with the distance to the hyperplane above 1.7 from the putative non drug target dataset

No.	Swiss-Prot AC	Protein Name	Distance
1	Q9NPG1	Frizzled-3	2.425
2	Q96PD7	Diacylglycerol O-acyltransferase 2	2.383
3	O75084	Frizzled-7	2.301
4	Q99835	Smoothened homolog	2.200
5	Q3MIR4	Cell cycle control protein 50B	2.184
6	Q14332	Frizzled-2	2.126
7	Q9HBY0	NADPH oxidase 3	2.040
8	Q5HYA8	Meckelin	2.037
9	Q9NYG2	Palmitoyltransferase ZDHHC3	2.020
10	Q96MH6	Transmembrane protein 68	1.999
11	Q9UL01	Dermatan-sulfate epimerase	1.974
12	Q5SY80	Uncharacterized protein C1orf101	1.914
13	Q9BQ90	Kelch domain-containing protein 3	1.827
14	P20618	Proteasome subunit beta type 1	1.818
15	Q8IZU2	WD repeat protein 17	1.810
16	Q9ULV1	Frizzled-4	1.796
17	P54803	Galactocerebrosidase	1.790
18	Q9NV96	Cell cycle control protein 50A	1.777
19	P51674	Neuronal membrane glycoprotein M6-a	1.776
20	P35503	UDP-glucuronosyltransferase 1–3	1.769
21	Q99571	P2X purinoceptor 4	1.744
22	P53396	ATP-citrate synthase	1.735
23	P08237	6-phosphofructokinase, muscle type	1.724
24	O00144	Frizzled-9	1.722
25	O95498	Vascular non-inflammatory molecule 2	1.721
26	P09848	Lactase-phlorizin hydrolase	1.718
27	Q05BV3	Echinoderm microtubule-associated protein-like 5	1.709
28	Q96QE2	Proton myo-inositol cotransporter	1.708
29	Q9NPF4	Probable O-sialoglycoprotein endopeptidase	1.707
30	Q9UJ83	2-hydroxyacyl-CoA lyase 1	1.704

Although many of predicted potential drug targets by this method have gained much interests in current research, whether a predicted drug target will finally become a true therapeutic drug target still needs more experimental test. Despite of that, these examples validate the effectiveness and predictive power of the present method. Meanwhile, it also indicates that this method can provide useful information in novel drug target identification and validation.

Besides the advantages discussed above, this method employed simple physicochemical properties based on primary protein sequence to construct the SVM model, which runs very fast even on a personal computer. However, the limitations of the current method should also be discussed. Firstly, only human proteins are covered by this method since the drug targets from other pathogens such as fungi, bacteria and viruses are not included, considering the difference between organisms and different mechanisms of function; secondly, only protein drug targets are taken into account, other types of targets such as DNA, RNA and protein-protein interfaces are beyond of the scope of the present study. The current method can be extended to predict these types of drug targets in the future when enough data are available.

## Conclusion

In the present study, a drug target prediction method based on support vector machine has been developed. Independent of homology annotation (or domain containing) and protein 3D structures, the current method employed a set of features of physicochemical properties directly calculated from primary protein sequence. The method can successfully distinguish known drug targets from putative non drug targets at an accuracy of 84% in 10-fold cross-validation test.

The model was further applied to four larger datasets with proteins in the drug-target families, research drug targets, research drug-target family members and putative non drug targets. Detailed analysis of the predicted drug targets with examples proves the effectiveness and predictive power of the model built. This method can be used in novel drug target identification and validation at large scale.

## Method

### Datasets

186 targets of approved oral small-molecular drugs were taken as the positive dataset in this study, which were recently revised by Overington et al[2]. The drug targets are listed in Additional file 2 as supplementary information. As there was no verified non drug target data readily available, we constructed a putative non drug-target dataset, following the way in the previous work[16], where the main idea was to remove possible drug targets and their protein family according to current knowledge (refer to Figure 4 for the relationship of different datasets used in this study).

The non drug target data was built by first retrieving 16,267 human-origin protein sequences from Swiss-Prot and eliminating the 186 drug target proteins and those in the relevant families. The 186 oral small-molecular drug targets covered 142 protein families in Pfam database [21], which contained 3,444 protein sequences. Therefore, only 12,823 protein sequences were remained after this step.

Then, 3,065 proteins of research drug target proteins of human origin from TTD[9] and DrugBank[10] and their relevant family members were also excluded. At the end, 9,758 protein sequences remained in the putative non drug-target dataset. It should be noted the protein sequences in the putative non drug-target dataset are not validated non drug-target proteins, which only means that till now no drug target (families) and research target (families) are found in this dataset. In fact, it will be of great value for drug target identification if novel drug targets or target families could be identified among this dataset. However, although novel drug targets might exist in this dataset, the chance is pretty low. Therefore, without substantially reducing SVM prediction performance, we randomly selected a certain number of protein sequences from this dataset to construct the negative dataset. The training and testing sets were constructed by the following procedure:

• Step 0: 186 targets of approved oral small-molecule drug targets were used as the positive data; the same number of proteins randomly selected from putative non drug-target dataset was taken as the negative data. There were a total of 372 proteins in this dataset.

• Step 1: 80% of samples (149 positive, 149 negative) from the dataset generated in step 0 were selected as training set (1). 10-fold cross-validation was applied when training the model.

• Step 2: The other 20% samples were used as blind test.

The procedures above were repeated for five times.

Considering the diversity of the putative non drug-target dataset, 186 proteins might not cover the entire "non drug-target space". Therefore, to better sampling the non drug-target space, we added more putative non drug-target proteins into the training set to improve sampling. The procedure was exactly the same as that mentioned above, except that the size of the negative dataset became larger.

• Step 0: 186 target proteins of approved oral small-molecule drug targets were still taken as the positive data; 372, 744, 1116, 1488, 1860 non drug-target proteins were randomly extracted from putative non drug-target dataset, which formed five negative sets with different sizes. Then, the size ratios of the positive set and the negative sets were 1:2, 1:4, 1:6, 1:8 and1:10, respectively.

• Training set: 80% of the positive set (149 drug-target proteins), combined with 80% of each negative set (298, 595, 893, 1190, 1490 non drug-target proteins) were used to construct another five different training sets which were referred as training set (2) – (6), respectively. Similarly, 10-fold cross-validation was applied to each of these datasets.

• Testing set: The rest of 20 % of positive data (37 drug target proteins) and each negative data (74, 149, 223, 298 and 370) were used for blind testing.

Each of the procedures was also repeated for five times.

To apply the model to the prediction of the larger dataset, four additional datasets were constructed. The relationship of these datasets is illustrated in Figure 4.

• **Dataset I: **Drug-target family. This dataset contained 3,444 human origin proteins from 142 drug target families. Here, protein family in Pfam database was defined based on domain affiliations or sequence clustering[16,21]. Proteins in drug target family are likely to be drug targets, but which needs further experimental validation.

• **Dataset II: **Research drug target. 371 research drug targets of human origin were selected from TTD[9] and DrugBank[10], and the other research drug targets which belong to drug target families were excluded. Note that here "research drug targets" refers to two groups of proteins, one is the proteins which have been used to develop drugs in experimental studies but have not been approved, the other group is the proteins which although were assigned as successful drug targets annotated by the databases, but not confirmed by the recently revised drug target list of Overington et al.

• **Dataset III: **Research drug target family. Based on dataset II, research drug targets and their related family members were included in this dataset, which contained 3,065 proteins.

• **Dataset IV: **Putative non-drug target. This dataset was the entire "putative non drug-target" dataset mentioned above, which consisted of 9,758 human origin proteins with drug targets and their families as well as research drug targets and their families removed.

### Features of the protein sequence

The feature set of a protein sequence was selected according to the previous studies[16,32,33]. Using a vector to represent a protein sequence, the percentage composition of the 20 amino acid residues formed the first 20 dimensions.

In addition, other six physicochemical properties were calculated, including hydrophobicity, polarity, polarizability, solvent accessibility and normalized *van der *Waals volume (Table 1). For each of these physicochemical properties, feature vectors were extracted from the primary sequence based on three descriptors: "Composition", composition percentage of three constituents (eg. polar, neutral and hydrophobic residues in hydrophobicity), which occupied three dimensions; "Transition", the transition frequencies (polar to neutral, neutral to hydrophobic, etc.), which occupied another three dimensions; and "Distribution", the distribution pattern of constituents (where the first residue of a given constituent was located, and where the 25%, 50%, 75%, and 100% of the constituent were contained), which occupied 15 dimensions (each constituent has five dimensions). Overall, each of these physicochemical properties occupied 21 dimensions. The complete feature vector consisted of 146 dimension elements.

### Feature selection with F-score

F-score is implemented as a tool in Libsvm package[20], which is a simple, intuitive method to evaluate the discrimination of two sets. Here, Given training vector *x*_*k*_, *k *= *1*,.., *m*, if the number of positive and negative instances are *n*_+ _and *n*_-_, respectively, then the F-score of the *i*th feature is defined as:

F(i)≡(x¯i(+)−x¯i)2+(x¯i(−)−x¯i)21n+−1∑k=1n+(xk,i(+)−x¯i(+))2+1n−−1∑k=1n−(xk,i(−)−x¯i(−))2
 MathType@MTEF@5@5@+=feaafiart1ev1aaatCvAUfKttLearuWrP9MDH5MBPbIqV92AaeXatLxBI9gBaebbnrfifHhDYfgasaacH8akY=wiFfYdH8Gipec8Eeeu0xXdbba9frFj0=OqFfea0dXdd9vqai=hGuQ8kuc9pgc9s8qqaq=dirpe0xb9q8qiLsFr0=vr0=vr0dc8meaabaqaciaacaGaaeqabaqabeGadaaakeaacqWGgbGrcqGGOaakcqWGPbqAcqGGPaqkcqGHHjIUdaWcaaqaaiabcIcaOiqbdIha4zaaraWaa0baaSqaaiabdMgaPbqaaiabcIcaOiabgUcaRiabcMcaPaaakiabgkHiTiqbdIha4zaaraWaaSbaaSqaaiabdMgaPbqabaGccqGGPaqkdaahaaWcbeqaaiabikdaYaaakiabgUcaRiabcIcaOiqbdIha4zaaraWaa0baaSqaaiabdMgaPbqaaiabcIcaOiabgkHiTiabcMcaPaaakiabgkHiTiqbdIha4zaaraWaaSbaaSqaaiabdMgaPbqabaGccqGGPaqkdaahaaWcbeqaaiabikdaYaaaaOqaamaalaaabaGaeGymaedabaGaemOBa42aaSbaaSqaaiabgUcaRaqabaGccqGHsislcqaIXaqmaaWaaabCaeaacqGGOaakcqWG4baEdaqhaaWcbaGaem4AaSMaeiilaWIaemyAaKgabaGaeiikaGIaey4kaSIaeiykaKcaaOGaeyOeI0IafmiEaGNbaebadaqhaaWcbaGaemyAaKgabaGaeiikaGIaey4kaSIaeiykaKcaaOGaeiykaKYaaWbaaSqabeaacqaIYaGmaaGccqGHRaWkdaWcaaqaaiabigdaXaqaaiabd6gaUnaaBaaaleaacqGHsislaeqaaOGaeyOeI0IaeGymaedaamaaqahabaGaeiikaGIaemiEaG3aa0baaSqaaiabdUgaRjabcYcaSiabdMgaPbqaaiabcIcaOiabgkHiTiabcMcaPaaakiabgkHiTiqbdIha4zaaraWaa0baaSqaaiabdMgaPbqaaiabcIcaOiabgkHiTiabcMcaPaaakiabcMcaPmaaCaaaleqabaGaeGOmaidaaaqaaiabdUgaRjabg2da9iabigdaXaqaaiabd6gaUnaaBaaameaacqGHsislaeqaaaqdcqGHris5aaWcbaGaem4AaSMaeyypa0JaeGymaedabaGaemOBa42aaSbaaWqaaiabgUcaRaqabaaaniabggHiLdaaaaaa@8B53@

where x¯i,x¯i(+),x¯i(−)
 MathType@MTEF@5@5@+=feaafiart1ev1aaatCvAUfKttLearuWrP9MDH5MBPbIqV92AaeXatLxBI9gBaebbnrfifHhDYfgasaacH8akY=wiFfYdH8Gipec8Eeeu0xXdbba9frFj0=OqFfea0dXdd9vqai=hGuQ8kuc9pgc9s8qqaq=dirpe0xb9q8qiLsFr0=vr0=vr0dc8meaabaqaciaacaGaaeqabaqabeGadaaakeaacuWG4baEgaqeamaaBaaaleaacqWGPbqAaeqaaOGaeiilaWIafmiEaGNbaebadaqhaaWcbaGaemyAaKgabaGaeiikaGIaey4kaSIaeiykaKcaaOGaeiilaWIafmiEaGNbaebadaqhaaWcbaGaemyAaKgabaGaeiikaGIaeyOeI0IaeiykaKcaaaaa@3CFD@ are the average of the *i*th feature of the whole, positive, and negative datasets, respectively; x¯k,i(+)
 MathType@MTEF@5@5@+=feaafiart1ev1aaatCvAUfKttLearuWrP9MDH5MBPbIqV92AaeXatLxBI9gBaebbnrfifHhDYfgasaacH8akY=wiFfYdH8Gipec8Eeeu0xXdbba9frFj0=OqFfea0dXdd9vqai=hGuQ8kuc9pgc9s8qqaq=dirpe0xb9q8qiLsFr0=vr0=vr0dc8meaabaqaciaacaGaaeqabaqabeGadaaakeaacuWG4baEgaqeamaaDaaaleaacqWGRbWAcqGGSaalcqWGPbqAaeaacqGGOaakcqGHRaWkcqGGPaqkaaaaaa@3498@ is the *i*th feature of the *k*th positive instance, and x¯k,i(−)
 MathType@MTEF@5@5@+=feaafiart1ev1aaatCvAUfKttLearuWrP9MDH5MBPbIqV92AaeXatLxBI9gBaebbnrfifHhDYfgasaacH8akY=wiFfYdH8Gipec8Eeeu0xXdbba9frFj0=OqFfea0dXdd9vqai=hGuQ8kuc9pgc9s8qqaq=dirpe0xb9q8qiLsFr0=vr0=vr0dc8meaabaqaciaacaGaaeqabaqabeGadaaakeaacuWG4baEgaqeamaaDaaaleaacqWGRbWAcqGGSaalcqWGPbqAaeaacqGGOaakcqGHsislcqGGPaqkaaaaaa@34A3@ is the *i*th feature of the *k*th negative instance. The larger the F-score is, the more likely discriminative this feature is. Therefore, we used this score as a feature selection criterion. In combination with support vector machine, in each round, selecting a possible threshold, the features with F-scores above the threshold were taken for training. This procedure was repeated until the best results were found.

### Support vector machine

The implementation of support vector machine used here is Libsvm[20]. Three different kernel functions, linear kernel, polynomial kernel and radial basis function (RBF) were evaluated in turn. According to the machine learning theory[34], an optimal hyperplane will be drawn by SVM model, in order to separate positive samples from negative ones. The distance to the hyperplane is related to the confidence of a prediction. Therefore, the distance from each sample to the hyperplane was employed to predict the drug target likeness of a protein.

### Performance evaluation

The performance of each model was evaluated with an *n*-fold cross-validation test. In the cross-validation test, the entire data set was shuffled and split into *n *folds. Each fold was used in turn for testing and the remaining part (n-1 folds) was used for training. The sensitivity (*Q*p), specificity (*Q*n) and overall accuracy (*Q*a) were used to measure the accuracy of positive prediction, negative prediction and the overall accuracy of the model [35], respectively.

*Q*_*p *_= *TP*/(*TP *+ *FN*)

*Q*_*n *_= *TN*/(*TN *+ *FP*)

*Q*_*a *_= (*TP *+ *TN*)/(*TP *+ *TN *+ *FP *+ *FN*)

Here, TP, TN, FP and FN represent true positives, true negatives, false positives and false negatives, respectively. In general, the overall accuracy *Q*a is always used to measure the predictive power of a model.

## Competing interests

The author(s) declares that there are no competing interests.

## Authors' contributions

The basic idea was conceived by LL. The implementation was developed by QL. Both authors have read and approved the final manuscript.

## Supplementary Material

Additional file 1 A total of 1,383 predicted drug targets from putative non drug target dataset. Total 1,383 potential novel drug targets predicted by current method. The Swiss-Prot ID and protein name with the distance score was listed in it. Click here for file

Additional file 2 186 targets of FDA-approved oral small-molecular drugs. Total 186 targets of FDA-approved oral small-molecular drugs. The SwissProt ID and protein name was in it. Click here for file
