# Supplementary material for: Prediction of potential drug targets based on simple sequence properties
Source: BMC Bioinformatics. 2007 Sep 20;8:353. doi: 10.1186/1471-2105-8-353 (PMC2082046; doi:10.1186/1471-2105-8-353)
Supplement: Additional file 1 — A total of 1,383 predicted drug targets from putative non drug target dataset. Total 1,383 potential novel drug targets predicted by current method. The Swiss-Prot ID and protein name with the distance score was listed in it. [file 1471-2105-8-353-S1.pdf]

Table S1. A total of 1,383 predicted drug targets from putative non drug-target dataset

No.	Swiss-Prot AC	Distance	Protein Name
1	Q9NPG1	2.4258	Frizzled-3
2	Q96PD7	2.3836	Diacylglycerol O-acyltransferase 2
3	O75084	2.3010	Frizzled-7
4	Q99835	2.2005	Smoothened homolog
5	Q3MIR4	2.1848	Cell cycle control protein 50B
6	Q14332	2.1263	Frizzled-2
7	Q9HBY0	2.0400	NADPH oxidase 3
8	Q5HYA8	2.0372	Meckelin
9	Q9NYG2	2.0207	Palmitoyltransferase ZDHHC3
10	Q96MH6	1.9998	Transmembrane protein 68
11	Q9UL01	1.9747	Dermatan-sulfate epimerase
12	Q5SY80	1.9144	Uncharacterized protein C1orf101
13	Q9BQ90	1.8275	Kelch domain-containing protein 3
14	P20618	1.8185	Proteasome subunit beta type 1
15	Q8IZU2	1.8101	WD repeat protein 17
16	Q9ULV1	1.7965	Frizzled-4
17	P54803	1.7904	Galactocerebrosidase
18	Q9NV96	1.7773	Cell cycle control protein 50A
19	P51674	1.7762	Neuronal membrane glycoprotein M6-a
20	P35503	1.7696	UDP-glucuronosyltransferase 1-3
21	Q99571	1.7440	P2X purinoceptor 4
22	P53396	1.7350	ATP-citrate synthase
23	P08237	1.7246	6-phosphofructokinase, muscle type
24	O00144	1.7221	Frizzled-9
25	O95498	1.7210	Vascular non-inflammatory molecule 2
26	P09848	1.7182	Lactase-phlorizin hydrolase
27	Q05BV3	1.7095	Echinoderm microtubule-associated protein-like 5
28	Q96QE2	1.7084	Proton myo-inositol cotransporter
29	Q9NPF4	1.7079	Probable O-sialoglycoprotein endopeptidase
30	Q9UJ83	1.7045	2-hydroxyacyl-CoA lyase 1
31	Q9H2Y9	1.6982	Solute carrier organic anion transporter family member 5A1
32	Q9BV94	1.6864	ER degradation-enhancing alpha-mannosidase-like 2
33	P46977	1.6756	Dolichyl-diphosphooligosaccharide--protein glycosyltransferase subunit STT3A
34	Q99523	1.6645	Sortilin
35	Q9UBM7	1.6615	7-dehydrocholesterol reductase
36	Q53GD3	1.6603	Choline transporter-like protein 4
37	P17858	1.6557	6-phosphofructokinase, liver type
38	P48651	1.6383	Phosphatidylserine synthase 1

No.	Swiss-Prot AC	Distance	Protein Name
39	O14494	1.6343	Lipid phosphate phosphohydrolase 1
40	P31040	1.6283	Succinate dehydrogenase [ubiquinone] flavoprotein subunit, mitochondrial
41	Q16348	1.6081	Oligopeptide transporter, kidney isoform
42	P61163	1.5989	Alpha-centractin
43	Q12794	1.5958	Hyaluronidase-1
44	Q86UL3	1.5880	1-acyl-sn-glycerol-3-phosphate acyltransferase zeta
45	P28072	1.5829	Proteasome subunit beta type 6
46	Q8IZU8	1.5797	Dermatan-sulfate epimerase-like protein
47	Q9UEF7	1.5699	Klotho
48	Q9Y519	1.5674	Putative MAP kinase-activating protein C22orf5
49	O95497	1.5589	Pantetheinase
50	P28070	1.5583	Proteasome subunit beta type 4
51	P33121	1.5578	Long-chain-fatty-acid--CoA ligase 1
52	Q8N1V2	1.5504	WD repeat protein 16
53	P35504	1.5477	UDP-glucuronosyltransferase 1-5
54	Q9H7T0	1.5349	Uncharacterized protein C14orf161
55	P42025	1.5327	Beta-centractin
56	Q86Z14	1.5133	Beta klotho
57	P26951	1.5126	Interleukin-3 receptor alpha chain
58	Q14264	1.5090	HERV-R_7q21.2 provirus ancestral Env polyprotein
59	Q6E213	1.5049	Acyl-CoA wax alcohol acyltransferase 2
60	Q01813	1.4999	6-phosphofructokinase type C
61	Q9NY84	1.4987	Vascular non-inflammatory molecule 3
62	O94923	1.4799	D-glucuronyl C5-epimerase
63	Q9P1W3	1.4673	Transmembrane protein 63C
64	O60656	1.4641	UDP-glucuronosyltransferase 1-9
65	P68133	1.4630	Actin, alpha skeletal muscle
66	Q9HCC0	1.4622	Methylcrotonoyl-CoA carboxylase beta chain, mitochondrial
67	Q9NUN5	1.4600	LMBR1 domain-containing protein 1
68	P19224	1.4574	UDP-glucuronosyltransferase 1-6
69	Q96S06	1.4567	Transmembrane protein 112
70	Q9BZW5	1.4387	Transmembrane 6 superfamily member 1
71	P23378	1.4355	Glycine dehydrogenase [decarboxylating], mitochondrial
72	Q8NDN9	1.4279	RCC1 and BTB domain-containing protein 1
73	P68032	1.4232	Actin, alpha cardiac muscle 1
74	P56373	1.4173	P2X purinoceptor 3
75	Q9HCG7	1.4124	Non-lysosomal glucosylceramidase
76	P63267	1.4095	Actin, gamma-enteric smooth muscle
77	P11168	1.4055	Solute carrier family 2, facilitated glucose transporter member 2
78	Q8WUJ3	1.4037	Protein KIAA1199
79	Q9NR16	1.3906	Scavenger receptor cysteine-rich type 1 protein M160
80	Q15269	1.3882	Periodic tryptophan protein 2 homolog
81	Q13467	1.3860	Frizzled-5

No.	Swiss-Prot AC	Distance	Protein Name
82	Q15124	1.3765	Phosphoglucomutase-like protein 5
83	Q6ICH7	1.3658	Aspartate beta-hydroxylase domain-containing protein 2
84	Q8NDV1	1.3555	Alpha-N-acetylgalactosaminide alpha-2,6-sialyltransferase 3
85	O95199	1.3511	RCC1 and BTB domain-containing protein 2
86	Q6UWM7	1.3508	Lactase-like protein
87	Q13510	1.3500	Acid ceramidase
88	Q9H3G5	1.3415	Probable serine carboxypeptidase CPVL
89	P62736	1.3412	Actin, aortic smooth muscle
90	P51575	1.3402	P2X purinoceptor 1
91	P35573	1.3400	Glycogen debranching enzyme
92	P40879	1.3327	Chloride anion exchanger
93	Q92508	1.3313	Protein FAM38A
94	Q9Y4X1	1.3282	UDP-glucuronosyltransferase 2A1
95	Q9NRY7	1.3282	Phospholipid scramblase 2
96	Q10981	1.3256	Galactoside 2-alpha-L-fucosyltransferase 2
97	P43251	1.3222	Biotinidase
98	P22310	1.3183	UDP-glucuronosyltransferase 1-4
99	P31512	1.3071	Dimethylaniline monooxygenase [N-oxide-forming] 4
100	Q9UKL4	1.3069	Gap junction alpha-9 protein
101	P04839	1.2978	Cytochrome b-245 heavy chain
102	Q9HAW9	1.2905	UDP-glucuronosyltransferase 1-8
103	P54840	1.2875	Glycogen [starch] synthase, liver
104	Q96H96	1.2851	Para-hydroxybenzoate--polyprenyltransferase, mitochondrial
105	Q12908	1.2842	Ileal sodium/bile acid cotransporter
106	O60353	1.2807	Frizzled-6
107	P49326	1.2779	Dimethylaniline monooxygenase [N-oxide-forming] 5
108	Q9HAW7	1.2747	UDP-glucuronosyltransferase 1-7
109	Q9BZ23	1.2739	Pantothenate kinase 2, mitochondrial
110	Q9UKU0	1.2736	Long-chain-fatty-acid--CoA ligase 6
111	P53621	1.2680	Coatomer subunit alpha
112	P13866	1.2657	Sodium/glucose cotransporter 1
113	Q9P0L9	1.2649	Polycystic kidney disease 2-like 1 protein
114	Q9NS93	1.2629	Transmembrane 7 superfamily member 3
115	Q9ULC5	1.2595	Long-chain-fatty-acid--CoA ligase 5
116	O75841	1.2562	Uroplakin-1b
117	Q9HAW8	1.2506	UDP-glucuronosyltransferase 1-10
118	Q96PL2	1.2487	Beta-tectorin
119	Q8IUH2	1.2390	Protein CREG2
120	Q96PD6	1.2371	2-acylglycerol O-acyltransferase 1
121	Q9NUB1	1.2340	Acetyl-coenzyme A synthetase 2-like, mitochondrial
122	P60709	1.2286	Actin, cytoplasmic 1
123	Q969U6	1.2283	F-box/WD repeat protein 5
124	Q9Y4P1	1.2246	Cysteine protease ATG4B

No.	Swiss-Prot AC	Distance	Protein Name
125	Q9UP38	1.2180	Frizzled-1
126	Q9ULW8	1.2162	Protein-arginine deiminase type-3
127	Q86UP9	1.2148	Lipoma HMGIC fusion partner-like 3 protein
128	Q8N4S9	1.2107	MARVEL domain-containing protein 2
129	Q7Z304	1.2083	MAM domain-containing protein 2
130	Q9NPC4	1.2082	Lactosylceramide 4-alpha-galactosyltransferase
131	P46059	1.2028	Oligopeptide transporter, small intestine isoform
132	P08236	1.1988	Beta-glucuronidase
133	Q8NHU2	1.1956	Uncharacterized protein C20orf26
134	Q8N653	1.1898	Leucine-zipper-like transcriptional regulator 1
135	O14975	1.1892	Very-long-chain acyl-CoA synthetase
136	Q6ZRH7	1.1865	Uncharacterized protein C19orf15
137	Q11203	1.1845	CMP-N-acetylneuraminate-beta-1,4-galactoside alpha-2,3- sialyltransferase
138	Q99487	1.1833	Platelet-activating factor acetylhydrolase 2, cytoplasmic
139	Q9NRK6	1.1804	ATP-binding cassette sub-family B member 10, mitochondrial
140	Q504Q3	1.1783	PAB-dependent poly(A)-specific ribonuclease subunit 2
141	Q96G23	1.1781	LAG1 longevity assurance homolog 2
142	Q8TCJ2	1.1780	Dolichyl-diphosphooligosaccharide--protein glycosyltransferase subunit STT3B
143	P35080	1.1728	Profilin-2
144	Q14108	1.1668	Lysosome membrane protein 2
145	O43520	1.1614	Probable phospholipid-transporting ATPase IC
146	Q93099	1.1578	Homogentisate 1,2-dioxygenase
147	O60423	1.1545	Probable phospholipid-transporting ATPase IK
148	Q9BYC2	1.1514	Succinyl-CoA:3-ketoacid-coenzyme A transferase 2, mitochondrial
149	Q9H227	1.1505	Cytosolic beta-glucosidase
150	Q92604	1.1505	Acyl-CoA:lysophosphatidylglycerol acyltransferase 1
151	Q8N8Q8	1.1503	Inner membrane protein COX18, mitochondrial
152	Q9NXL6	1.1477	SID1 transmembrane family member 1
153	Q8IWA5	1.1474	Choline transporter-like protein 2
154	Q9ULW2	1.1351	Frizzled-10
155	Q16518	1.1324	Retinal pigment epithelium-specific 65 kDa protein
156	Q9NZC3	1.1322	Glycerophosphodiester phosphodiesterase 1
157	O00462	1.1272	Beta-mannosidase
158	Q9NY91	1.1271	Low affinity sodium-glucose cotransporter
159	P20933	1.1271	N(4)-(beta-N-acetylglucosaminyl)-L-asparaginase
160	Q6ZWT7	1.1247	Membrane-bound O-acyltransferase domain-containing protein 2
161	Q9Y5S8	1.1215	NADPH oxidase homolog 1
162	Q9HA77	1.1205	Probable cysteinyl-tRNA synthetase, mitochondrial
163	Q8IU99	1.1193	Protein FAM26C
164	P40126	1.1190	L-dopachrome tautomerase
165	P63261	1.1189	Actin, cytoplasmic 2
166	Q8NEZ3	1.1171	WD repeat protein 19
167	Q8NBJ9	1.1134	SID1 transmembrane family member 2

No.	Swiss-Prot AC	Distance	Protein Name
168	P05108	1.1121	Cytochrome P450 11A1, mitochondrial
169	Q3SYC2	1.1120	2-acylglycerol O-acyltransferase 2
170	Q96KN2	1.1076	Beta-Ala-His dipeptidase
171	P16930	1.1035	Fumarylacetoacetase
172	Q8TDB8	1.1025	Solute carrier family 2, facilitated glucose transporter member 14
173	P13807	1.0975	Glycogen [starch] synthase, muscle
174	Q96GX1	1.0973	Tectonic-2
175	Q15436	1.0884	Protein transport protein Sec23A
176	Q9Y4E6	1.0841	WD repeat protein 7
177	P29144	1.0770	Tripeptidyl-peptidase 2
178	Q02083	1.0756	N-acylethanolamine-hydrolyzing acid amidase
179	Q9HAY6	1.0726	Beta,beta-carotene 15,15'-monooxygenase
180	P53794	1.0718	Sodium/myo-inositol cotransporter
181	Q9NWZ5	1.0713	Uridine/cytidine kinase-like 1
182	Q9H1J5	1.0712	Protein Wnt-8a
183	Q8WVQ1	1.0634	Soluble calcium-activated nucleotidase 1
184	Q5GH73	1.0632	XK-related protein 6
185	Q9UQF0	1.0625	HERV-W_7q21.2 provirus ancestral Env polyprotein
186	Q96DB2	1.0588	Histone deacetylase 11
187	Q10570	1.0565	Cleavage and polyadenylation specificity factor subunit 1
188	P06756	1.0554	Integrin alpha-V
189	Q9UKT5	1.0528	F-box only protein 4
190	Q8N7A1	1.0506	Kelch domain-containing protein 1
191	Q92611	1.0490	ER degradation-enhancing alpha-mannosidase-like 1
192	Q9Y2J8	1.0483	Protein-arginine deiminase type-2
193	Q9NYB5	1.0461	Solute carrier organic anion transporter family member 1C1
194	P01031	1.0440	Complement C5
195	Q01344	1.0383	Interleukin-5 receptor alpha chain
196	Q5GLZ8	1.0380	Probable E3 ubiquitin-protein ligase HERC4
197	Q8TDG2	1.0365	Actin-related protein T1
198	O60502	1.0352	Bifunctional protein NCOAT
199	Q9H0V1	1.0325	Transmembrane protein 168
200	Q96DT6	1.0313	Cysteine protease ATG4C
201	P61566	1.0297	HERV-K_22q11.21 provirus ancestral Env polyprotein
202	Q9H9Y6	1.0274	DNA-directed RNA polymerase I subunit RPA2
203	Q96MB7	1.0268	Uncharacterized protein C11orf77
204	Q5T3F8	1.0268	Transmembrane protein 63B
205	Q9NR71	1.0225	Neutral ceramidase
206	Q5TEA3	1.0224	Uncharacterized protein C20orf194
207	P54802	1.0122	Alpha-N-acetylglucosaminidase
208	P55809	1.0105	Succinyl-CoA:3-ketoacid-coenzyme A transferase 1, mitochondrial
209	Q5HY92	1.0091	Fidgetin
210	Q6IA86	1.0065	Elongator complex protein 2

No.	Swiss-Prot AC	Distance	Protein Name
211	Q9Y4K0	1.0052	Lysyl oxidase homolog 2
212	Q15070	1.0025	Inner membrane protein OXA1L, mitochondrial
213	Q9NXK6	1.0023	Membrane progestin receptor gamma
214	Q6ZPD8	1.0005	Diacylglycerol O-acyltransferase 2-like protein 6
215	P15509	0.9999	Granulocyte-macrophage colony-stimulating factor receptor alpha chain
216	P17948	0.9996	Vascular endothelial growth factor receptor 1
217	Q9HCJ1	0.9990	Progressive ankylosis protein homolog
218	Q8WU66	0.9983	Protein TSPEAR
219	Q86VF5	0.9962	2-acylglycerol O-acyltransferase 3
220	Q9UPU3	0.9959	VPS10 domain-containing receptor SorCS3
221	O94955	0.9949	Rho-related BTB domain-containing protein 3
222	Q9H3D4	0.9935	Tumor protein p73-like
223	O60637	0.9874	Tetraspanin-3
224	Q9HC07	0.9850	Transmembrane protein 165
225	Q14117	0.9849	Dihydropyrimidinase
226	Q9P2H3	0.9826	Intraflagellar transport 80 homolog
227	Q8N5B7	0.9821	LAG1 longevity assurance homolog 5
228	P61158	0.9815	Actin-like protein 3
229	Q0VDG4	0.9790	Secernin-3
230	Q8TCT9	0.9778	Minor histocompatibility antigen H13
231	Q92959	0.9771	Solute carrier organic anion transporter family member 2A1
232	P49768	0.9737	Presenilin-1
233	Q7Z3F1	0.9717	Integral membrane protein GPR155
234	Q01740	0.9671	Dimethylaniline monooxygenase [N-oxide-forming] 1
235	Q8NFT2	0.9627	Metalloreductase STEAP2
236	Q96EY1	0.9622	DnaJ homolog subfamily A member 3, mitochondrial
237	Q99572	0.9611	P2X purinoceptor 7
238	Q9NRZ5	0.9603	1-acyl-sn-glycerol-3-phosphate acyltransferase delta
239	Q9HAC7	0.9590	Uncharacterized protein C7orf10
240	Q13093	0.9565	Platelet-activating factor acetylhydrolase
241	P23470	0.9540	Receptor-type tyrosine-protein phosphatase gamma
242	Q9H553	0.9508	Alpha-1,3-mannosyltransferase ALG2
243	Q16880	0.9494	2-hydroxyacylsphingosine 1-beta-galactosyltransferase
244	Q5GH72	0.9470	XK-related protein 7
245	Q9NQT6	0.9423	Fascin-3
246	Q8NDY3	0.9418	[Protein ADP-ribosylarginine] hydrolase-like protein 1
247	P61568	0.9408	HERV-K_1p13.3 provirus ancestral Env polyprotein
248	Q86Y01	0.9401	Protein deltex-1
249	Q8NFZ6	0.9369	Vomeronasal type-1 receptor 2
250	O00767	0.9362	Acyl-CoA desaturase
251	Q9NZ32	0.9354	Actin-related protein 10
252	O60774	0.9326	Putative dimethylaniline monooxygenase [N-oxide-forming] 6
253	Q9Y236	0.9306	Oxidative stress induced growth inhibitor 2

No.	Swiss-Prot AC	Distance	Protein Name
254	Q9H300	0.9289	Presenilins-associated rhomboid-like protein, mitochondrial
255	Q06430	0.9273	N-acetyllactosaminide beta-1,6-N-acetylglucosaminyl-transferase
256	Q9H0B8	0.9270	Cysteine-rich secretory protein LCCL domain-containing 2
257	Q12907	0.9218	Vesicular integral-membrane protein VIP36
258	Q9BYD9	0.9216	Actin-related protein M1
259	P51993	0.9211	Alpha-(1,3)-fucosyltransferase
260	Q9H1C4	0.9199	UNC93 homolog B1
261	Q96Q42	0.9194	Alsin
262	O94886	0.9190	Transmembrane protein 63A
263	Q15437	0.9164	Protein transport protein Sec23B
264	Q70EL3	0.9160	Inactive ubiquitin carboxyl-terminal hydrolase 50
265	Q9NPL8	0.9146	Uncharacterized protein C3orf1
266	O94901	0.9134	Sad1/unc-84 protein-like 1
267	Q8WW27	0.9120	Putative C-
268	O15033	0.9112	Protein KIAA0317
269	Q8NCS7	0.9093	Choline transporter-like protein 5
270	Q5TDP6	0.9091	Glutamate--ammonia ligase domain-containing protein 1
271	Q9NRM6	0.9076	Interleukin-17 receptor B
272	Q96N76	0.9032	Probable urocanate hydratase
273	Q96QG7	0.9024	Myotubularin-related protein 9
274	Q5JPE7	0.9016	Nodal modulator 2
275	P15104	0.9011	Glutamine synthetase
276	P04180	0.9011	Phosphatidylcholine-sterol acyltransferase
277	Q9P2L0	0.8992	WD repeat protein 35
278	P61567	0.8909	HERV-K_1q22 provirus ancestral Env polyprotein
279	Q15034	0.8908	Probable E3 ubiquitin-protein ligase HERC3
280	Q9NRF8	0.8846	CTP synthase 2
281	Q9NR34	0.8831	Mannosyl-oligosaccharide 1,2-alpha-mannosidase IC
282	Q9NZI5	0.8813	Grainyhead-like protein 1 homolog
283	O75694	0.8808	Nuclear pore complex protein Nup155
284	Q5TGI0	0.8790	Uncharacterized protein C6orf168
285	Q86TX2	0.8774	Acyl-coenzyme A thioesterase 1
286	P58215	0.8712	Lysyl oxidase homolog 3
287	Q9Y315	0.8711	Putative deoxyribose-phosphate aldolase
288	Q9HAT2	0.8710	Sialate O-acetylesterase
289	P31513	0.8692	Dimethylaniline monooxygenase [N-oxide-forming] 3
290	Q16549	0.8688	Proprotein convertase subtilisin/kexin type 7
291	P10267	0.8685	HERV-K_5q33.3 provirus ancestral Env polyprotein
292	Q8TF62	0.8627	Probable phospholipid-transporting ATPase IM
293	Q8WTR4	0.8617	Glycerophosphodiester phosphodiesterase domain-containing protein 5
294	P11498	0.8607	Pyruvate carboxylase, mitochondrial
295	Q8NDH3	0.8584	Probable aminopeptidase NPEPL1
296	O96005	0.8570	Cleft lip and palate transmembrane protein 1

No.	Swiss-Prot AC	Distance	Protein Name
297	Q9NRZ7	0.8561	1-acyl-sn-glycerol-3-phosphate acyltransferase gamma
298	P22309	0.8541	UDP-glucuronosyltransferase 1-1
299	Q9BWM7	0.8492	Sideroflexin-3
300	Q8TED0	0.8465	U3 small nucleolar RNA-associated protein 15 homolog
301	P16671	0.8459	Platelet glycoprotein 4
302	Q8TE04	0.8452	Pantothenate kinase 1
303	P22760	0.8408	Arylacetamide deacetylase
304	Q9Y276	0.8402	Mitochondrial chaperone BCS1
305	O75356	0.8400	Ectonucleoside triphosphate diphosphohydrolase 5
306	Q9GZN1	0.8391	Actin-related protein 6
307	Q8NDZ0	0.8375	Uncharacterized protein CXorf20
308	Q5T197	0.8363	DC-STAMP domain-containing protein 1
309	Q9UMX1	0.8345	Suppressor of fused homolog
310	Q93052	0.8344	Lipoma-preferred partner
311	Q9Y4W6	0.8334	AFG3-like protein 2
312	Q00403	0.8330	Transcription initiation factor IIB
313	Q9BUB7	0.8308	Transmembrane protein 70
314	Q8TAF8	0.8287	Lipoma HMGIC fusion partner-like 5 protein
315	P39656	0.8270	Dolichyl-diphosphooligosaccharide--protein glycosyltransferase 48 kDa subunit
316	Q8N4N3	0.8255	Kelch repeat and BTB domain-containing protein C16orf44
317	Q9NPH5	0.8235	NADPH oxidase 4
318	P14672	0.8223	Solute carrier family 2, facilitated glucose transporter member 4
319	Q969W1	0.8220	Probable palmitoyltransferase ZDHHC16
320	P30876	0.8203	DNA-directed RNA polymerase II 140 kDa polypeptide
321	P19526	0.8192	Galactoside 2-alpha-L-fucosyltransferase 1
322	Q8N3J5	0.8188	Protein phosphatase 1K, mitochondrial
323	Q96NU7	0.8168	Probable imidazolonepropionase
324	Q6P1M0	0.8159	Long-chain fatty acid transport protein 4
325	P69849	0.8156	Nodal modulator 3
326	Q99538	0.8147	Legumain
327	P30520	0.8143	Adenylosuccinate synthetase isozyme 2
328	Q9HB40	0.8129	Retinoid-inducible serine carboxypeptidase
329	Q6IA69	0.8118	Glutamine-dependent NAD(+) synthetase
330	Q96SE0	0.8116	Abhydrolase domain-containing protein 1
331	Q01581	0.8108	Hydroxymethylglutaryl-CoA synthase, cytoplasmic
332	P18074	0.8096	TFIIH basal transcription factor complex helicase subunit
333	Q8NCC3	0.8077	1-O-acylceramide synthase
334	O95436	0.8067	Sodium-dependent phosphate transport protein 2B
335	Q92995	0.8053	Ubiquitin carboxyl-terminal hydrolase 13
336	P31146	0.8053	Coronin-1A
337	Q8NB12	0.8039	SET and MYND domain-containing protein 1
338	Q9HCJ5	0.8020	Zinc finger SWIM domain-containing protein 6 (Fragment)
339	P12277	0.8011	Creatine kinase B-type

No.	Swiss-Prot AC	Distance	Protein Name
340	Q03154	0.8001	Aminoacylase-1
341	Q9NRQ2	0.7985	Phospholipid scramblase 4
342	Q15155	0.7985	Nodal modulator 1
343	Q9UMR5	0.7983	Lysosomal thioesterase PPT2
344	Q5GH76	0.7977	XK-related protein 4
345	Q9BY64	0.7961	UDP-glucuronosyltransferase 2B28
346	Q9BZB8	0.7960	Cytoplasmic polyadenylation element-binding protein 1
347	P48506	0.7933	Glutamate--cysteine ligase catalytic subunit
348	O14986	0.7929	Phosphatidylinositol-4-phosphate 5-kinase type-1 beta
349	O14925	0.7901	Mitochondrial import inner membrane translocase subunit Tim23
350	Q5VUY2	0.7889	Arylacetamide deacetylase-like 4
351	Q8NFZ0	0.7888	F-box only protein 18
352	P14679	0.7875	Tyrosinase
353	Q9HDB8	0.7866	HERV-K_3q12.3 provirus ancestral Env polyprotein
354	O75355	0.7864	Ectonucleoside triphosphate diphosphohydrolase 3
355	Q9HD40	0.7863	O-phosphoseryl-tRNA(Sec) selenium transferase
356	P27544	0.7862	LAG1 longevity assurance homolog 1
357	O43556	0.7862	Epsilon-sarcoglycan
358	P13726	0.7859	Tissue factor
359	Q9H5I5	0.7858	Protein FAM38B
360	P60509	0.7855	HERV-R(b)_3p24.3 provirus ancestral Env polyprotein
361	Q9UIB8	0.7809	SLAM family member 5
362	O75844	0.7808	CAAX prenyl protease 1 homolog
363	P46597	0.7741	Hydroxyindole O-methyltransferase
364	P29122	0.7739	Proprotein convertase subtilisin/kexin type 6
365	Q93086	0.7703	P2X purinoceptor 5
366	Q9NNW5	0.7700	WD repeat protein 6
367	Q9Y2K9	0.7691	Syntaxin-binding protein 5-like
368	Q9UJM8	0.7686	Hydroxyacid oxidase 1
369	Q15393	0.7682	Splicing factor 3B subunit 3
370	P78406	0.7654	mRNA export factor
371	Q2TBA0	0.7636	Kelch repeat and BTB domain-containing protein 5
372	O00391	0.7635	Sulfhydryl oxidase 1
373	P50395	0.7629	Rab GDP dissociation inhibitor beta
374	Q13085	0.7623	Acetyl-CoA carboxylase 1
375	Q93100	0.7618	Phosphorylase b kinase regulatory subunit beta
376	Q9NX78	0.7613	Uncharacterized protein C14orf101
377	Q9H7P6	0.7598	Protein FAM125B
378	Q53S58	0.7592	Transmembrane protein 177
379	P60508	0.7578	HERV-FRD_6p24.1 provirus ancestral Env polyprotein
380	Q9UI40	0.7564	Sodium/potassium/calcium exchanger 2
381	P13639	0.7498	Elongation factor 2
382	Q9UJ37	0.7476	Alpha-N-acetylgalactosaminide alpha-2,6-sialyltransferase 2

No.	Swiss-Prot AC	Distance	Protein Name
383	Q9BR76	0.7466	Coronin-1B
384	Q9UBV4	0.7446	Protein Wnt-16
385	O95954	0.7437	Formimidoyltransferase-cyclodeaminase
386	Q9UQ90	0.7373	Paraplegin
387	Q99519	0.7352	Sialidase-1
388	Q6PCE3	0.7349	Phosphoglucomutase-2-like 1
389	Q9H568	0.7331	Actin-like protein 8
390	Q96BD0	0.7322	Solute carrier organic anion transporter family member 4A1
391	Q6ZV70	0.7303	LanC-like protein 3
392	Q9UQ49	0.7284	Sialidase-3
393	Q9H3N1	0.7281	Thioredoxin domain-containing protein 1
394	O42043	0.7258	HERV-K_1q23.3 provirus ancestral Env polyprotein
395	Q8N6M5	0.7247	Probable allantoicase
396	Q8TBH0	0.7229	Arrestin domain-containing protein 2
397	Q9BTX1	0.7226	Nucleoporin NDC1
398	Q9BXR0	0.7215	Queuine tRNA-ribosyltransferase
399	O43820	0.7208	Hyaluronidase-3
400	Q9BS26	0.7204	Thioredoxin domain-containing protein 4
401	O75310	0.7200	UDP-glucuronosyltransferase 2B11
402	P49753	0.7170	Acyl-coenzyme A thioesterase 2
403	Q96LU5	0.7167	Mitochondrial inner membrane protease subunit 1
404	Q93063	0.7138	Exostosin-2
405	O71037	0.7122	HERV-K_19q12 provirus ancestral Env polyprotein
406	P60507	0.7111	HERV-F(c)1_Xq21.33 provirus ancestral Env polyprotein
407	Q7Z403	0.7106	Transmembrane channel-like protein 6
408	O43688	0.7103	Lipid phosphate phosphohydrolase 2
409	Q9Y2E5	0.7099	Epididymis-specific alpha-mannosidase
410	Q86WB7	0.7095	UNC93 homolog A
411	Q8N9L9	0.7066	Acyl-coenzyme A thioesterase 4
412	Q9Y2E6	0.7050	Protein deltex-4
413	Q99627	0.7036	COP9 signalosome complex subunit 8
414	P01033	0.7027	Metalloproteinase inhibitor 1
415	Q8NCR0	0.7025	UDP-GalNAc:beta-1,3-N-acetylgalactosaminyltransferase 2
416	Q9NQT5	0.6989	Exosome complex exonuclease RRP40
417	Q10713	0.6977	Mitochondrial-processing peptidase alpha subunit, mitochondrial
418	O94805	0.6969	Actin-like protein 6B
419	Q92828	0.6962	Coronin-2A
420	Q8NCG7	0.6946	Sn1-specific diacylglycerol lipase beta
421	O75439	0.6946	Mitochondrial-processing peptidase subunit beta, mitochondrial
422	Q8IYD2	0.6945	Kelch domain-containing protein 8A
423	Q96EY5	0.6919	Protein FAM125A
424	Q8NBX0	0.6907	Probable saccharopine dehydrogenase
425	O15230	0.6898	Laminin subunit alpha-5

No.	Swiss-Prot AC	Distance	Protein Name
426	Q9Y2T3	0.6895	Guanine deaminase
427	O60701	0.6870	UDP-glucose 6-dehydrogenase
428	Q11128	0.6865	Alpha-(1,3)-fucosyltransferase
429	Q5JSJ4	0.6857	Protein DDX26B
430	O14495	0.6849	Lipid phosphate phosphohydrolase 3
431	Q8NFH4	0.6845	Nucleoporin Nup37
432	Q13228	0.6844	Selenium-binding protein 1
433	P46976	0.6843	Glycogenin-1
434	Q58A45	0.6835	PAB-dependent poly(A)-specific ribonuclease subunit 3
435	P49915	0.6830	GMP synthase [glutamine-hydrolyzing]
436	O75306	0.6817	NADH dehydrogenase [ubiquinone] iron-sulfur protein 2, mitochondrial
437	Q9NUQ2	0.6809	1-acyl-sn-glycerol-3-phosphate acyltransferase epsilon
438	Q9H9S3	0.6793	Protein transport protein Sec61 subunit alpha isoform 2
439	P16662	0.6787	UDP-glucuronosyltransferase 2B7
440	Q96G03	0.6786	Phosphoglucomutase-2
441	P48200	0.6786	Iron-responsive element-binding protein 2
442	Q6P5S2	0.6773	Uncharacterized protein C6orf58
443	P46019	0.6770	Phosphorylase b kinase regulatory subunit alpha, liver isoform
444	P49821	0.6763	NADH dehydrogenase [ubiquinone] flavoprotein 1, mitochondrial
445	P09544	0.6752	Protein Wnt-2
446	P33908	0.6749	Mannosyl-oligosaccharide 1,2-alpha-mannosidase IA
447	P25788	0.6746	Proteasome subunit alpha type 3
448	Q6PCB7	0.6726	Long-chain fatty acid transport protein 1
449	Q96S97	0.6725	Myeloid-associated differentiation marker
450	O95158	0.6725	Neurexophilin-4
451	Q5VW38	0.6722	Protein GPR107
452	Q92747	0.6718	Actin-related protein 2/3 complex subunit 1A
453	Q9Y6N1	0.6710	Cytochrome c oxidase assembly protein COX11, mitochondrial
454	P11169	0.6694	Solute carrier family 2, facilitated glucose transporter member 3
455	Q9HCN3	0.6683	Transmembrane protein 8
456	O94823	0.6653	Probable phospholipid-transporting ATPase VB
457	P58499	0.6652	Protein FAM3B
458	Q9NVE7	0.6649	Pantothenate kinase 4
459	P53677	0.6635	AP-3 complex subunit mu-2
460	Q96LB8	0.6626	Peptidoglycan recognition protein I-beta
461	O00116	0.6613	Alkyldihydroxyacetonephosphate synthase, peroxisomal
462	Q9UKJ8	0.6610	ADAM 21
463	A0AVT1	0.6603	Ubiquitin-activating enzyme E1-like protein 2
464	Q9HBJ8	0.6580	Collectrin
465	Q9Y471	0.6563	Cytidine monophosphate-N-acetylneuraminic acid hydroxylase-like protein
466	Q969X6	0.6558	Cirhin
467	Q8NFF2	0.6545	Sodium/potassium/calcium exchanger 4
468	Q9H0C3	0.6542	Transmembrane protein 117

No.	Swiss-Prot AC	Distance	Protein Name
469	Q6NUS6	0.6535	Tectonic-3
470	P46721	0.6530	Solute carrier organic anion transporter family member 1A2
471	Q5T9L3	0.6474	Integral membrane protein GPR177
472	Q7Z3C6	0.6471	Autophagy-related protein 9A
473	Q15084	0.6466	Protein disulfide-isomerase A6
474	P37287	0.6446	Phosphatidylinositol N-acetylglucosaminyltransferase subunit A
475	Q9BTV4	0.6440	Transmembrane protein 43
476	O75387	0.6439	Large neutral amino acids transporter small subunit 3
477	Q96RR1	0.6437	Twinkle protein, mitochondrial
478	Q969X1	0.6435	Transmembrane BAX inhibitor motif-containing protein 1
479	Q9NUE0	0.6433	Palmitoyltransferase ZDHHC18
480	Q8N142	0.6431	Adenylosuccinate synthetase isozyme 1
481	P01024	0.6420	Complement C3
482	Q9UJ70	0.6408	N-acetylglucosamine kinase
483	Q5JRX3	0.6406	Presequence protease, mitochondrial
484	Q9P241	0.6384	Probable phospholipid-transporting ATPase VD
485	Q16658	0.6374	Fascin
486	O00763	0.6373	Acetyl-CoA carboxylase 2
487	Q8NAT1	0.6370	Uncharacterized glycosyltransferase AGO61
488	Q96TA2	0.6351	ATP-dependent metalloprotease YME1L1
489	Q9NXF8	0.6350	Palmitoyltransferase ZDHHC7
490	Q14627	0.6349	Interleukin-13 receptor alpha-2 chain
491	Q9H0W9	0.6345	Ester hydrolase C11orf54
492	Q6MZZ7	0.6343	Calpain-13
493	Q16635	0.6316	Tafazzin
494	Q92185	0.6299	Alpha-N-acetylneuraminide alpha-2,8-sialyltransferase
495	Q9GZS1	0.6297	DNA-directed RNA polymerase I-associated factor 53 kDa subunit
496	Q13347	0.6281	Eukaryotic translation initiation factor 3 subunit 2
497	Q9H9G7	0.6275	Eukaryotic translation initiation factor 2C 3
498	P05166	0.6264	Propionyl-CoA carboxylase beta chain, mitochondrial
499	Q13126	0.6242	S-methyl-5-thioadenosine phosphorylase
500	Q9UGQ3	0.6220	Solute carrier family 2, facilitated glucose transporter member 6
501	Q9ULV4	0.6218	Coronin-1C
502	P32019	0.6209	Type II inositol-1,4,5-trisphosphate 5-phosphatase
503	O96019	0.6186	Actin-like protein 6A
504	Q9NR19	0.6180	Acetyl-coenzyme A synthetase, cytoplasmic
505	Q86SQ6	0.6170	Probable G-protein coupled receptor 123
506	P17540	0.6166	Creatine kinase, sarcomeric mitochondrial
507	Q8WU17	0.6165	RING finger protein 139
508	Q9H4M9	0.6147	EH domain-containing protein 1
509	Q8NFG4	0.6136	Folliculin
510	Q9Y573	0.6133	Actin-binding protein IPP
511	Q9NX77	0.6127	HERV-K_16p3.3 provirus ancestral Env polyprotein

No.	Swiss-Prot AC	Distance	Protein Name
512	Q15029	0.6125	116 kDa U5 small nuclear ribonucleoprotein component
513	Q9UKM7	0.6118	Endoplasmic reticulum mannosyl-oligosaccharide 1,2-alpha-mannosidase
514	Q6ZMG9	0.6110	LAG1 longevity assurance homolog 6
515	O95620	0.6103	tRNA-dihydrouridine synthase 4-like
516	Q53R12	0.6095	Transmembrane 4 L6 family member 20
517	Q86VB7	0.6088	Scavenger receptor cysteine-rich type 1 protein M130
518	Q8IXB1	0.6058	DnaJ homolog subfamily C member 10
519	P11245	0.6054	Arylamine N-acetyltransferase 2
520	Q9NRX5	0.6049	Serine incorporator 1
521	Q8N465	0.6048	D-2-hydroxyglutarate dehydrogenase, mitochondrial
522	Q9NXC2	0.6025	Glucose-fructose oxidoreductase domain-containing protein 1
523	Q8TAA9	0.6000	Vang-like protein 1
524	O94956	0.5991	Solute carrier organic anion transporter family member 2B1
525	Q8TEQ6	0.5987	Gem-associated protein 5
526	Q9H4F1	0.5978	Alpha-N-acetyl-neuraminyl-2,3-beta-galactosyl-1,3-N-acetyl- galactosaminide alpha
527	Q86W33	0.5978	Integral membrane protein GPR175
528	Q9HC73	0.5977	Cytokine receptor-like factor 2
529	Q96GX9	0.5977	APAF1-interacting protein
530	O43301	0.5975	Heat shock 70 kDa protein 12A
531	O75165	0.5973	DnaJ homolog subfamily C member 13
532	Q9BYV7	0.5966	Beta,beta-carotene 9',10'-dioxygenase
533	Q5VSG8	0.5961	Glycoprotein endo-alpha-1,2-mannosidase-like protein
534	Q8N442	0.5956	GTP-binding protein GUF1 homolog
535	P38567	0.5949	Hyaluronidase PH-20
536	Q69384	0.5945	HERV-K_7p22.1 provirus ancestral Env polyprotein
537	Q7Z7L7	0.5929	Zyg-11 protein homolog
538	O60678	0.5927	Protein arginine N-methyltransferase 3
539	Q7Z4I7	0.5912	LIM and senescent cell antigen-like-containing domain protein 2
540	O15350	0.5909	Tumor protein p73
541	P53370	0.5897	Nucleoside diphosphate-linked moiety X motif 6
542	P30566	0.5881	Adenylosuccinate lyase
543	P61619	0.5857	Protein transport protein Sec61 subunit alpha isoform 1
544	O95045	0.5835	Uridine phosphorylase 2
545	Q96LB9	0.5819	Peptidoglycan recognition protein I-alpha
546	Q04721	0.5819	Neurogenic locus notch homolog protein 2
547	O95859	0.5816	Tetraspanin-12
548	Q8WZA1	0.5785	Protein O-linked-mannose beta-1,2-N-acetylglucosaminyltransferase 1
549	Q92562	0.5776	SAC domain-containing protein 3
550	P35606	0.5769	Coatomer subunit beta'
551	Q9BYW1	0.5752	Solute carrier family 2, facilitated glucose transporter member 11
552	Q8N4M1	0.5751	Choline transporter-like protein 3
553	Q8IVQ6	0.5736	Probable palmitoyltransferase ZDHHC21
554	O00754	0.5730	Lysosomal alpha-mannosidase

No.	Swiss-Prot AC	Distance	Protein Name
555	Q6TGC4	0.5717	Protein-arginine deiminase type-6
556	Q9NZN4	0.5709	EH domain-containing protein 2
557	Q8TBR7	0.5702	Protein FAM57A
558	P15144	0.5689	Aminopeptidase N
559	Q8NI60	0.5671	Chaperone-activity of bc1 complex-like, mitochondrial
560	Q5T6V5	0.5656	Uncharacterized protein C9orf64
561	Q9P2K2	0.5641	Thioredoxin domain-containing protein KIAA1344
562	Q96RP9	0.5629	Elongation factor G 1, mitochondrial
563	Q902F8	0.5626	HERV-K_8p23.1 provirus ancestral Env polyprotein
564	Q9NW08	0.5610	DNA-directed RNA polymerase III subunit 127.6 kDa polypeptide
565	Q8N9V3	0.5607	WD repeat, SAM and U-box domain-containing protein 1
566	P23435	0.5602	Cerebellin-1
567	Q9UK96	0.5593	F-box only protein 10
568	Q9UMX9	0.5585	Membrane-associated transporter protein
569	Q8IWV8	0.5569	E3 ubiquitin-protein ligase UBR2
570	Q9Y227	0.5567	Ectonucleoside triphosphate diphosphohydrolase 4
571	Q8NE79	0.5539	Blood vessel epicardial substance
572	P63135	0.5532	HERV-K_1q22 provirus ancestral Pol protein
573	Q5T5C0	0.5521	Syntaxin-binding protein 5
574	Q9NX05	0.5504	UPF0318 protein FAM120C
575	Q15042	0.5503	Rab3 GTPase-activating protein catalytic subunit
576	Q8NES3	0.5501	Beta-1,3-N-acetylglucosaminyltransferase lunatic fringe
577	Q6ZMY6	0.5442	WD repeat protein 88
578	Q96NB2	0.5416	Sideroflexin-2
579	O75648	0.5409	tRNA
580	Q6UWF7	0.5406	Protein FAM55D
581	Q8NBS3	0.5404	Sodium bicarbonate transporter-like protein 11
582	Q96GQ5	0.5391	UPF0420 protein C16orf58
583	Q9H2C0	0.5388	Gigaxonin
584	P36537	0.5358	UDP-glucuronosyltransferase 2B10
585	P41252	0.5322	Isoleucyl-tRNA synthetase, cytoplasmic
586	Q9Y2U9	0.5319	Kelch domain-containing protein 2
587	Q8N122	0.5310	Regulatory-associated protein of mTOR
588	P17812	0.5309	CTP synthase 1
589	P36382	0.5307	Gap junction alpha-5 protein
590	O14525	0.5306	Astrotactin-1
591	Q9Y263	0.5305	Phospholipase A-2-activating protein
592	Q6P4A8	0.5295	LAMA-like protein 1
593	Q4G0P3	0.5287	Hydrocephalus-inducing protein homolog
594	O00442	0.5282	RNA 3'-terminal phosphate cyclase
595	Q9BSA9	0.5274	Transmembrane protein 175
596	Q9NZV5	0.5272	Selenoprotein N
597	Q16831	0.5264	Uridine phosphorylase 1

No.	Swiss-Prot AC	Distance	Protein Name
598	Q14118	0.5264	Dystroglycan
599	P10266	0.5262	HERV-K_5q33.3 provirus ancestral Pol protein
600	O43506	0.5261	ADAM 20
601	Q902F9	0.5260	HERV-K_19p13.11 provirus ancestral Env polyprotein
602	Q9Y5V3	0.5252	Melanoma-associated antigen D1
603	Q9NRY6	0.5238	Phospholipid scramblase 3
604	Q96N67	0.5234	Dedicator of cytokinesis protein 7
605	Q9H0C5	0.5224	BTB/POZ domain-containing protein 1
606	Q8IYS1	0.5209	Aminoacylase 1-like protein 2
607	Q8NEL9	0.5202	Probable phospholipase DDHD1
608	Q9GZV3	0.5192	High-affinity choline transporter 1
609	P12532	0.5190	Creatine kinase, ubiquitous mitochondrial
610	Q9UL03	0.5188	Integrator complex subunit 6
611	Q969X5	0.5176	Endoplasmic reticulum-Golgi intermediate compartment protein 1
612	Q8NCQ5	0.5163	F-box only protein 15
613	Q14410	0.5159	Glycerol kinase, testis specific 2
614	Q9P0J1	0.5145	[Pyruvate dehydrogenase [lipoamide]]-phosphatase 1, mitochondrial
615	Q86W34	0.5141	Archaemetzincin-2
616	Q9P2S5	0.5136	WD repeat protein 8
617	O60337	0.5132	E3 ubiquitin-protein ligase MARCH6
618	Q9Y6L6	0.5122	Solute carrier organic anion transporter family member 1B1
619	Q16706	0.5104	Alpha-mannosidase 2
620	Q9UKN8	0.5095	General transcription factor 3C polypeptide 4
621	P61565	0.5095	HERV-K_12q14.1 provirus ancestral Env polyprotein
622	Q92874	0.5094	Deoxyribonuclease I-like 2
623	Q9UGI0	0.5080	Zinc finger Ran-binding domain-containing protein 1
624	Q86UB9	0.5079	Transmembrane protein 135
625	Q96F25	0.5072	UDP-N-acetylglucosamine transferase subunit ALG14 homolog
626	Q96FA3	0.5065	Protein pellino homolog 1
627	Q3T906	0.5040	N-acetylglucosamine-1-phosphotransferase subunits alpha/beta
628	Q8TDJ6	0.5039	Protein DmX-like 2
629	Q9BYT9	0.5038	Transmembrane protein 16C
630	Q6P2Q9	0.5036	Pre-mRNA-processing-splicing factor 8
631	Q9NXS3	0.5030	Kelch-like protein 28
632	Q6P1X5	0.5023	Transcription initiation factor TFIID subunit 2
633	Q9NTJ4	0.5014	Alpha-mannosidase 2C1
634	Q9H223	0.5001	EH domain-containing protein 4
635	Q8NEZ5	0.5000	F-box only protein 22
636	P55895	0.4995	V(D)J recombination-activating protein 2
637	O14672	0.4988	ADAM 10
638	P31639	0.4978	Sodium/glucose cotransporter 2
639	P07737	0.4976	Profilin-1
640	Q9NYV8	0.4963	Taste receptor type 2 member 14

No.	Swiss-Prot AC	Distance	Protein Name
641	Q9Y5K1	0.4946	Meiotic recombination protein SPO11
642	Q6UX65	0.4939	Transmembrane protein 77
643	Q8WUE5	0.4928	Uncharacterized protein CXorf48
644	Q9Y2E4	0.4922	Disco-interacting protein 2 homolog C
645	Q9UBU6	0.4917	Protein FAM8A1
646	Q86SG7	0.4907	Lysozyme g-like protein 2
647	Q9NRM0	0.4901	Solute carrier family 2, facilitated glucose transporter member 9
648	Q8WWI5	0.4898	Choline transporter-like protein 1
649	P18440	0.4890	Arylamine N-acetyltransferase 1
650	Q9Y3I0	0.4885	UPF0027 protein C22orf28
651	Q6P1M3	0.4883	Lethal(2) giant larvae protein homolog 2
652	O95704	0.4875	Amyloid beta A4 protein-binding family B member 3
653	Q9NYQ3	0.4866	Hydroxyacid oxidase 2
654	Q9NYU2	0.4864	UDP-glucose:glycoprotein glucosyltransferase 1
655	Q96B86	0.4863	Repulsive guidance molecule A
656	Q6ZUI0	0.4845	Protein FAM79B
657	Q9BTE6	0.4831	Alanyl-tRNA synthetase domain-containing protein 1
658	Q8N766	0.4824	Uncharacterized protein KIAA0090
659	Q9H336	0.4818	Cysteine-rich secretory protein LCCL domain-containing 1
660	Q9NRR4	0.4816	Ribonuclease 3
661	Q9UHL4	0.4808	Dipeptidyl-peptidase 2
662	O94953	0.4794	JmjC domain-containing histone demethylation protein 3B
663	Q9UG56	0.4789	Phosphatidylserine decarboxylase proenzyme
664	Q14194	0.4788	Dihydropyrimidinase-related protein 1
665	Q15125	0.4783	3-beta-hydroxysteroid-Delta(8),Delta(7)-isomerase
666	P32929	0.4780	Cystathionine gamma-lyase
667	Q6P4A7	0.4770	Sideroflexin-4
668	P61421	0.4767	Vacuolar ATP synthase subunit d 1
669	Q63HN8	0.4763	RING finger protein 213
670	P46020	0.4760	Phosphorylase b kinase regulatory subunit alpha, skeletal muscle isoform
671	Q9H920	0.4755	RING finger protein 121
672	Q9UKH3	0.4754	HERV-K_6q14.1 provirus ancestral Env polyprotein
673	Q9NV23	0.4747	S-acyl fatty acid synthase thioesterase, medium chain
674	Q9Y2P4	0.4724	Long-chain fatty acid transport protein 6
675	Q99518	0.4722	Dimethylaniline monooxygenase [N-oxide-forming] 2
676	Q92542	0.4705	Nicastrin
677	P51654	0.4696	Glypican-3
678	O95631	0.4695	Netrin-1
679	Q9NY64	0.4694	Solute carrier family 2, facilitated glucose transporter member 8
680	Q96RQ3	0.4689	Methylcrotonoyl-CoA carboxylase subunit alpha, mitochondrial
681	Q8IWR0	0.4678	Zinc finger CCCH domain-containing protein 7A
682	P56703	0.4673	Proto-oncogene protein Wnt-3
683	Q9BZH6	0.4666	Bromodomain and WD repeat domain-containing protein 2

No.	Swiss-Prot AC	Distance	Protein Name
684	Q71RS6	0.4665	Sodium/potassium/calcium exchanger 5
685	Q99447	0.4662	Ethanolamine-phosphate cytidylyltransferase
686	P00966	0.4642	Argininosuccinate synthase
687	Q6P587	0.4634	Fumarylacetoacetate hydrolase domain-containing protein 1
688	Q14849	0.4618	StAR-related lipid transfer protein 3
689	Q63HM2	0.4613	Uncharacterized protein C14orf135
690	P49641	0.4612	Alpha-mannosidase IIx
691	Q9HBG6	0.4607	Intraflagellar transport 122 homolog
692	Q6RW13	0.4591	Type-1 angiotensin II receptor-associated protein
693	Q9ULX6	0.4581	A-kinase anchor protein 8-like
694	Q96L92	0.4577	Sorting nexin-27
695	P33992	0.4575	DNA replication licensing factor MCM5
696	O95486	0.4575	Protein transport protein Sec24A (Fragment)
697	P04066	0.4562	Tissue alpha-L-fucosidase
698	Q92824	0.4557	Proprotein convertase subtilisin/kexin type 5
699	Q969S9	0.4537	Elongation factor G 2, mitochondrial
700	Q9H598	0.4505	Vesicular inhibitory amino acid transporter
701	Q08380	0.4499	Galectin-3-binding protein
702	Q9HB63	0.4492	Netrin-4
703	Q9UM07	0.4487	Protein-arginine deiminase type-4
704	Q9BSN7	0.4482	Claudin-like protein 24
705	Q8IVU3	0.4480	Probable E3 ubiquitin-protein ligase HERC6
706	Q7Z7J7	0.4477	Lipoma HMGIC fusion partner-like 4 protein
707	P11166	0.4474	Solute carrier family 2, facilitated glucose transporter member 1
708	O95452	0.4473	Gap junction beta-6 protein
709	P58743	0.4445	Prestin
710	P28065	0.4442	Proteasome subunit beta type 9
711	Q9H1P3	0.4441	Oxysterol-binding protein-related protein 2
712	Q96DT5	0.4433	Ciliary dynein heavy chain 11
713	Q8TCB0	0.4432	Interferon-induced protein 44
714	Q8TB22	0.4430	Spermatogenesis-associated protein 20
715	Q96Q91	0.4420	Anion exchange protein 4
716	Q96NS1	0.4418	Protein yippee-like 4
717	Q14156	0.4417	Protein EFR3-like
718	Q15654	0.4416	Thyroid receptor-interacting protein 6
719	P49588	0.4403	Alanyl-tRNA synthetase, cytoplasmic
720	A1L020	0.4386	RNA-binding protein MEX3A
721	Q8N4F7	0.4378	RING finger protein 175
722	Q96M94	0.4376	Kelch-like protein 15
723	P31150	0.4372	Rab GDP dissociation inhibitor alpha
724	Q86UW9	0.4370	Protein deltex-2
725	O95573	0.4363	Long-chain-fatty-acid--CoA ligase 3
726	P49961	0.4362	Ectonucleoside triphosphate diphosphohydrolase 1

No.	Swiss-Prot AC	Distance	Protein Name
727	Q96KP4	0.4343	Cytosolic non-specific dipeptidase
728	Q8TDN7	0.4323	Alkaline ceramidase 1
729	P25787	0.4278	Proteasome subunit alpha type 2
730	Q6J4K2	0.4271	Sodium/potassium/calcium exchanger 6
731	Q9NYC9	0.4245	Ciliary dynein heavy chain 9
732	O75808	0.4239	Calpain-15
733	O43684	0.4233	Mitotic checkpoint protein BUB3
734	O75795	0.4232	UDP-glucuronosyltransferase 2B17
735	Q86TL0	0.4208	Cysteine protease ATG4D
736	Q96HP0	0.4207	Dedicator of cytokinesis protein 6
737	P52701	0.4204	DNA mismatch repair protein MSH6
738	Q16394	0.4201	Exostosin-1
739	Q9NPB8	0.4193	Putative glycerophosphodiester phosphodiesterase 5
740	Q9Y606	0.4191	tRNA pseudouridine synthase A
741	Q6ZSI9	0.4179	Calpain-12
742	Q9NZB2	0.4176	UPF0318 protein FAM120A
743	O95772	0.4156	MLN64 N-terminal domain homolog
744	Q13496	0.4140	Myotubularin
745	Q9Y316	0.4124	Protein MEMO1
746	Q9NS86	0.4123	LanC-like protein 2
747	Q6ISB3	0.4120	Grainyhead-like protein 2 homolog
748	O00628	0.4110	Peroxisomal targeting signal 2 receptor
749	Q7Z401	0.4109	C-myc promoter-binding protein
750	Q96HA4	0.4101	Uncharacterized protein C1orf159
751	Q03167	0.4097	TGF-beta receptor type III
752	Q9NYP3	0.4089	Protein downstream neighbor of Son
753	Q5W0Z9	0.4082	Probable palmitoyltransferase ZDHHC20
754	P31153	0.4071	S-adenosylmethionine synthetase isoform type-2
755	Q9NP59	0.4067	Solute carrier family 40 member 1
756	Q15035	0.4065	Translocation-associated membrane protein 2
757	Q96S15	0.4064	WD repeat protein 24
758	Q96GW9	0.4064	Methionyl-tRNA synthetase, mitochondrial
759	Q9P258	0.4061	Protein RCC2
760	O75153	0.4042	Putative eukaryotic translation initiation factor 3 subunit
761	Q53H47	0.4028	Histone-lysine N-methyltransferase SETMAR
762	P16519	0.4021	Neuroendocrine convertase 2
763	Q68CP4	0.4018	Heparan-alpha-glucosaminide N-acetyltransferase
764	Q14703	0.3998	Membrane-bound transcription factor site-1 protease
765	O95528	0.3997	Solute carrier family 2, facilitated glucose transporter member 10
766	Q9H5V8	0.3987	CUB domain-containing protein 1
767	P10768	0.3973	S-formylglutathione hydrolase
768	Q9UMY4	0.3970	Sorting nexin-12
769	P21399	0.3969	Iron-responsive element-binding protein 1

No.	Swiss-Prot AC	Distance	Protein Name
770	Q8NBN3	0.3968	Transmembrane protein 87A
771	Q9P217	0.3963	Zinc finger SWIM domain-containing protein 5
772	Q8NFH5	0.3954	Nucleoporin NUP53
773	Q8N3G9	0.3929	Transmembrane protein 130
774	Q70JA7	0.3917	Chondroitin sulfate synthase 3
775	P58658	0.3913	Uncharacterized protein C21orf63
776	Q9Y312	0.3907	Uncharacterized protein C20orf4
777	Q9H9B4	0.3891	Sideroflexin-1
778	Q9H7M6	0.3890	Zinc finger SWIM domain-containing protein 4
779	P40121	0.3886	Macrophage-capping protein
780	Q96RD6	0.3884	Pannexin-2
781	Q9NPD5	0.3844	Solute carrier organic anion transporter family member 1B3
782	Q8TCE6	0.3842	Protein FAM45A
783	O75534	0.3834	Cold shock domain-containing protein E1
784	Q9UQ03	0.3830	Coronin-2B
785	P28749	0.3830	Retinoblastoma-like protein 1
786	Q9Y4P8	0.3796	WD repeat domain phosphoinositide-interacting protein 2
787	Q8WVR3	0.3790	Uncharacterized protein C7orf43
788	P36268	0.3784	Gamma-glutamyltransferase-like protein 2
789	Q9HAT8	0.3770	Protein pellino homolog 2
790	Q6QEF8	0.3770	Coronin-6
791	O60931	0.3738	Cystinosin
792	P54855	0.3732	UDP-glucuronosyltransferase 2B15
793	Q4KMQ2	0.3712	Transmembrane protein 16F
794	Q9UBV7	0.3706	Beta-1,4-galactosyltransferase 7
795	P54098	0.3695	DNA polymerase subunit gamma 1
796	Q9Y5Y7	0.3689	Lymphatic vessel endothelial hyaluronic acid receptor 1
797	P49842	0.3684	Serine/threonine-protein kinase 19
798	Q86X10	0.3680	Protein KIAA1219
799	Q5QJU3	0.3673	Alkaline ceramidase 2
800	O95377	0.3638	Gap junction beta-5 protein
801	P10619	0.3634	Lysosomal protective protein
802	Q9NQZ7	0.3620	Ectonucleoside triphosphate diphosphohydrolase 7
803	Q8WYK0	0.3608	Acyl-coenzyme A thioesterase 12
804	Q8WVP7	0.3596	Limb region 1 protein homolog
805	Q9H497	0.3593	Torsin-3A
806	Q6ZUX7	0.3588	Lipoma HMGIC fusion partner-like 2 protein
807	O95373	0.3583	Importin-7
808	Q9UBX8	0.3571	Beta-1,4-galactosyltransferase 6
809	Q9NY33	0.3561	Dipeptidyl-peptidase 3
810	P08034	0.3551	Gap junction beta-1 protein
811	Q70EK9	0.3548	Ubiquitin carboxyl-terminal hydrolase 51
812	O43761	0.3528	Synaptogyrin-3

No.	Swiss-Prot AC	Distance	Protein Name
813	Q86YT5	0.3522	Solute carrier family 13 member 5
814	Q6NSI4	0.3519	Uncharacterized protein CXorf57
815	P61160	0.3519	Actin-like protein 2
816	Q64LD2	0.3500	WD repeat protein 25
817	Q96B67	0.3499	Arrestin domain-containing protein 3
818	Q8IWA0	0.3495	WD repeat protein 75
819	Q8WTS6	0.3489	Histone-lysine N-methyltransferase, H3 lysine-4 specific SET7
820	Q9H488	0.3484	GDP-fucose protein O-fucosyltransferase 1
821	Q9P253	0.3478	Vacuolar protein sorting-associated protein 18 homolog
822	P36871	0.3473	Phosphoglucomutase-1
823	Q9BVG9	0.3463	Phosphatidylserine synthase 2
824	O43909	0.3463	Exostosin-like 3
825	Q9Y615	0.3447	Actin-like protein 7A
826	Q5VUG0	0.3435	Scm-like with four MBT domains protein 2
827	Q9UNX4	0.3423	WD repeat protein 3
828	Q9Y6I4	0.3419	Ubiquitin carboxyl-terminal hydrolase 3
829	O15162	0.3412	Phospholipid scramblase 1
830	Q9BTW9	0.3408	Tubulin-specific chaperone D
831	Q9H295	0.3401	Transmembrane 7 superfamily member 4
832	Q9Y3F4	0.3398	Serine-threonine kinase receptor-associated protein
833	Q9NZN3	0.3395	EH domain-containing protein 3
834	Q9NPA1	0.3393	Calcium-activated potassium channel subunit beta 3
835	Q8NFL0	0.3392	UDP-GlcNAc:betaGal beta-1,3-N-acetylglucosaminyltransferase 7
836	O60830	0.3388	Mitochondrial import inner membrane translocase subunit Tim17-B
837	Q9UHJ3	0.3384	Scm-like with four MBT domains protein 1
838	Q8WXF7	0.3370	Atlastin-1
839	Q8WUM9	0.3366	Sodium-dependent phosphate transporter 1
840	Q969R5	0.3362	Lethal(3)malignant brain tumor-like 2 protein
841	Q05996	0.3361	Zona pellucida sperm-binding protein 2
842	Q92935	0.3359	Exostosin-like 1
843	Q14202	0.3346	Zinc finger MYM-type protein 3
844	Q9Y296	0.3345	Trafficking protein particle complex subunit 4
845	Q9UIA9	0.3337	Exportin-7
846	Q5JST6	0.3337	EF-hand domain-containing family member C2
847	Q96HP8	0.3336	Transmembrane protein 176A
848	Q92643	0.3333	GPI-anchor transamidase
849	O15321	0.3331	Transmembrane 9 superfamily protein member 1
850	Q86WU2	0.3325	Probable D-lactate dehydrogenase, mitochondrial
851	Q9NQ90	0.3321	Transmembrane protein 16B
852	Q9ULK4	0.3318	CRSP complex subunit 3
853	Q9NV66	0.3312	tRNA wybutosine-synthesizing protein 1 homolog A
854	Q6ZVN8	0.3311	Hemojuvelin
855	Q8NE62	0.3303	Choline dehydrogenase, mitochondrial

No.	Swiss-Prot AC	Distance	Protein Name
856	Q5VUY0	0.3298	Arylacetamide deacetylase-like 3
857	O76036	0.3298	Natural cytotoxicity triggering receptor 1
858	Q9Y231	0.3295	Alpha-(1,3)-fucosyltransferase
859	Q5VT40	0.3287	Protein FAM78B
860	Q4G0Z9	0.3278	Uncharacterized protein C8orf45
861	O75592	0.3275	Probable E3 ubiquitin-protein ligase MYCBP2
862	P52797	0.3262	Ephrin-A3
863	O95352	0.3261	Autophagy-related protein 7
864	Q6UWU4	0.3257	Uncharacterized protein C6orf89
865	O75251	0.3256	NADH dehydrogenase [ubiquinone] iron-sulfur protein 7, mitochondrial
866	Q8IY47	0.3252	Kelch repeat and BTB domain-containing protein 2
867	P09001	0.3249	Mitochondrial 39S ribosomal protein L3
868	Q9BPU6	0.3247	Dihydropyrimidinase-related protein 5
869	P49447	0.3247	Cytochrome b561
870	Q9NVR2	0.3245	Integrator complex subunit 10
871	Q16842	0.3233	CMP-N-acetylneuraminate-beta-galactosamide-alpha-2,3-sialyltransferase
872	Q96PE3	0.3226	Type I inositol-3,4-bisphosphate 4-phosphatase
873	Q9UJW3	0.3224	DNA
874	O95985	0.3224	DNA topoisomerase 3-beta-1
875	O75503	0.3211	Ceroid-lipofuscinosis neuronal protein 5
876	Q9UL18	0.3207	Eukaryotic translation initiation factor 2C 1
877	Q9BVL4	0.3203	Selenoprotein O
878	O14735	0.3202	CDP-diacylglycerol--inositol 3-phosphatidyltransferase
879	Q6U841	0.3182	Sodium-driven chloride bicarbonate exchanger
880	Q08AF3	0.3169	Schlafen family member 5
881	Q8N239	0.3157	Kelch-like protein 34
882	Q9NX20	0.3156	39S ribosomal protein L16, mitochondrial
883	Q8IWV7	0.3143	E3 ubiquitin-protein ligase UBR1
884	O95163	0.3142	Elongator complex protein 1
885	O15547	0.3114	P2X purinoceptor 6
886	P51580	0.3112	Thiopurine S-methyltransferase
887	Q86YN1	0.3101	Dolichyldiphosphatase 1
888	Q2MV58	0.3093	Tectonic-1
889	P22314	0.3083	Ubiquitin-activating enzyme E1
890	P50443	0.3081	Sulfate transporter
891	P45974	0.3068	Ubiquitin carboxyl-terminal hydrolase 5
892	Q96FK6	0.3065	WD repeat protein 89
893	Q8IXV7	0.3046	Kelch domain-containing protein 8B
894	Q9ULE6	0.3043	Paladin
895	Q96MM6	0.3041	Heat shock 70 kDa protein 12B
896	Q9NTT1	0.3036	Putative ubiquitin-conjugating enzyme E2 D3-like protein
897	P24386	0.3033	Rab proteins geranylgeranyltransferase component A 1
898	Q6W3E5	0.3032	Glycerophosphodiester phosphodiesterase domain-containing protein 4

No.	Swiss-Prot AC	Distance	Protein Name
899	Q8N3I7	0.3025	Bardet-Biedl syndrome 5 protein
900	Q6NSW5	0.3023	Protein FAM45B
901	P42357	0.3022	Histidine ammonia-lyase
902	Q09328	0.3018	Alpha-1,6-mannosylglycoprotein 6-beta-N-acetylglucosaminyltransferase A
903	Q6ZTR5	0.3010	Uncharacterized protein CXorf22
904	Q9Y512	0.2998	Sorting and assembly machinery component 50 homolog
905	Q6UX01	0.2998	Protein LMBR1L
906	P08962	0.2991	CD63 antigen
907	Q58HT5	0.2979	Acyl-CoA wax alcohol acyltransferase 1
908	Q9H9S5	0.2977	Fukutin-related protein
909	P48165	0.2974	Gap junction alpha-8 protein
910	Q9UGK8	0.2967	Secretion-regulating guanine nucleotide exchange factor
911	Q13488	0.2955	Vacuolar proton translocating ATPase 116 kDa subunit a isoform 3
912	Q8NFK1	0.2950	Gap junction epsilon-1 protein
913	Q8TB61	0.2949	Adenosine 3'-phospho 5'-phosphosulfate transporter 1
914	P52948	0.2947	Nuclear pore complex protein Nup98-Nup96 [Contains: Nuclear pore complex protein Nu[illegible]
915	O94919	0.2939	Endonuclease domain-containing 1 protein
916	Q8IV20	0.2906	UPF0124 protein C13orf31
917	Q12891	0.2900	Hyaluronidase-2
918	P21217	0.2891	Galactoside 3(4)-L-fucosyltransferase
919	Q00266	0.2885	S-adenosylmethionine synthetase isoform type-1
920	P48059	0.2885	LIM and senescent cell antigen-like-containing domain protein 1
921	O00400	0.2883	Acetyl-coenzyme A transporter 1
922	Q8N8Y2	0.2881	Vacuolar ATP synthase subunit d 2
923	Q8NCI6	0.2868	Beta-galactosidase-1-like protein 3
924	P62253	0.2859	Ubiquitin-conjugating enzyme E2 G1
925	O43490	0.2832	Prominin-1
926	O95487	0.2826	Protein transport protein Sec24B
927	Q9NTQ9	0.2822	Gap junction beta-4 protein
928	Q8TAA3	0.2814	Proteasome subunit alpha type 7-like
929	Q9UIC8	0.2806	Leucine carboxyl methyltransferase 1
930	Q9BTY2	0.2794	Plasma alpha-L-fucosidase
931	P36957	0.2791	Dihydrolipoyllysine-residue succinyltransferase component of 2
932	Q969N2	0.2790	GPI transamidase component PIG-T
933	Q9NVE5	0.2789	Ubiquitin carboxyl-terminal hydrolase 40
934	P55160	0.2787	Nck-associated protein 1-like
935	P28347	0.2784	Transcriptional enhancer factor TEF-1
936	Q9P291	0.2782	Armadillo repeat-containing X-linked protein 1
937	Q14409	0.2772	Glycerol kinase, testis specific 1
938	Q9NZM6	0.2747	Polycystic kidney disease 2-like 2 protein
939	O60488	0.2737	Long-chain-fatty-acid--CoA ligase 4
940	Q9UNH6	0.2730	Sorting nexin-7
941	P51398	0.2730	Mitochondrial 28S ribosomal protein S29

No.	Swiss-Prot AC	Distance	Protein Name
942	Q9HCK5	0.2725	Eukaryotic translation initiation factor 2C 4
943	Q14689	0.2717	Disco-interacting protein 2 homolog A
944	Q6PI48	0.2714	Aspartyl-tRNA synthetase, mitochondrial
945	Q9NVX2	0.2712	Notchless protein homolog 1
946	O94855	0.2710	Protein transport protein Sec24D
947	Q8WY21	0.2708	VPS10 domain-containing receptor SorCS1
948	Q99798	0.2700	Aconitate hydratase, mitochondrial
949	Q9HCQ5	0.2697	Polypeptide N-acetylgalactosaminyltransferase 9
950	O76062	0.2695	Delta(14)-sterol reductase
951	P55259	0.2686	Pancreatic secretory granule membrane major glycoprotein GP2
952	Q9C0H5	0.2678	Protein KIAA1688
953	P29376	0.2675	Leukocyte tyrosine kinase receptor
954	Q14192	0.2667	Four and a half LIM domains protein 2
955	Q6ZTY8	0.2663	Putative uncharacterized protein C12orf63
956	Q9UBC3	0.2660	DNA
957	P50876	0.2659	Ubiquitin-conjugating enzyme 7-interacting protein 4
958	P51809	0.2632	Synaptobrevin-like protein 1
959	O43315	0.2632	Aquaporin-9
960	O14744	0.2628	Protein arginine N-methyltransferase 5
961	O43813	0.2626	LanC-like protein 1
962	O15527	0.2619	N-glycosylase/DNA lyase [Includes: 8-oxoguanine DNA glycosylase
963	O14841	0.2595	5-oxoprolinase
964	Q96PQ7	0.2594	Kelch-like protein 5
965	Q9HCL2	0.2591	Glycerol-3-phosphate acyltransferase, mitochondrial
966	O43286	0.2588	Beta-1,4-galactosyltransferase 5
967	Q92949	0.2585	Forkhead box protein J1
968	O60907	0.2582	F-box-like/WD repeat protein TBL1X
969	Q96JG8	0.2580	Melanoma-associated antigen D4
970	Q96GC9	0.2576	Transmembrane protein 49
971	O75562	0.2573	Protein HFSE-1
972	O75533	0.2573	Splicing factor 3B subunit 1
973	Q16342	0.2566	Programmed cell death protein 2
974	P42695	0.2562	Condensin-II complex subunit D3
975	P22083	0.2562	Alpha-(1,3)-fucosyltransferase
976	Q96J01	0.2555	THO complex subunit 3
977	Q8ND61	0.2555	Uncharacterized protein C3orf20
978	Q13505	0.2548	Metaxin-1
979	O75140	0.2545	DEP domain-containing protein 5
980	O15143	0.2541	Actin-related protein 2/3 complex subunit 1B
981	Q9H0R1	0.2517	Uncharacterized protein C14orf108
982	Q9UKG4	0.2508	Solute carrier family 13 member 4
983	Q9BUN8	0.2483	Derlin-1
984	P31930	0.2473	Ubiquinol-cytochrome-c reductase complex core protein 1, mitochondrial

No.	Swiss-Prot AC	Distance	Protein Name
985	Q8IUX1	0.2472	Transmembrane protein 126B
986	Q9BZW4	0.2461	Transmembrane 6 superfamily member 2
987	Q96JD4	0.2458	Uncharacterized protein C6orf114
988	Q32P44	0.2455	Echinoderm microtubule-associated protein-like 3
989	Q9P0N8	0.2454	E3 ubiquitin-protein ligase MARCH2
990	Q8N485	0.2447	Protein limb expression 1 homolog
991	Q09019	0.2432	Dystrophia myotonica WD repeat-containing protein
992	O60909	0.2431	Beta-1,4-galactosyltransferase 2
993	O14802	0.2431	DNA-directed RNA polymerase III largest subunit
994	Q96RY7	0.2425	Intraflagellar transport 140 homolog
995	Q7Z3K3	0.2422	Pogo transposable element with ZNF domain
996	Q9H6U8	0.2411	Alpha-1,2-mannosyltransferase ALG9
997	Q9HBK9	0.2409	Arsenite methyltransferase
998	Q96EP9	0.2403	Sodium/bile acid cotransporter 4
999	P78396	0.2400	Cyclin-A1
1000	Q9H3R0	0.2387	JmjC domain-containing histone demethylation protein 3C
1001	O14603	0.2384	PTPN13-like protein, Y-linked
1002	Q9BYK8	0.2383	Peroxisomal proliferator-activated receptor A-interacting complex 285 kDa protein
1003	Q9NRG4	0.2379	SET and MYND domain-containing protein 2
1004	Q00597	0.2368	Fanconi anemia group C protein
1005	Q5SGD2	0.2365	Protein phosphatase 1L
1006	P16279	0.2365	Beta-galactosidase-related protein
1007	Q9ULC6	0.2361	Protein-arginine deiminase type-1
1008	P52209	0.2357	6-phosphogluconate dehydrogenase, decarboxylating
1009	Q15334	0.2354	Lethal(2) giant larvae protein homolog 1
1010	Q99999	0.2334	Galactosylceramide sulfotransferase
1011	Q9Y6N6	0.2326	Laminin subunit gamma-3
1012	Q96BI3	0.2324	Gamma-secretase subunit APH-1A
1013	Q15392	0.2320	24-dehydrocholesterol reductase
1014	Q8WWT9	0.2317	Solute carrier family 13 member 3
1015	O75354	0.2279	Ectonucleoside triphosphate diphosphohydrolase 6
1016	Q9NX47	0.2276	E3 ubiquitin-protein ligase MARCH5
1017	Q5U4P2	0.2265	Aspartate beta-hydroxylase domain-containing protein 1
1018	P36969	0.2265	Phospholipid hydroperoxide glutathione peroxidase, mitochondrial
1019	Q8TD91	0.2257	Melanoma-associated antigen C3
1020	Q7Z3V4	0.2250	Ubiquitin-protein ligase E3B
1021	Q9Y4D2	0.2230	Sn1-specific diacylglycerol lipase alpha
1022	Q9Y2C4	0.2221	Endonuclease G-like 1
1023	Q96F07	0.2218	Cytoplasmic FMR1-interacting protein 2
1024	O00322	0.2214	Uroplakin-1a
1025	Q9NXE4	0.2196	Sphingomyelin phosphodiesterase 4
1026	Q8NE00	0.2189	Transmembrane protein 104
1027	Q9HBE1	0.2184	POZ-, AT hook-, and zinc finger-containing protein 1

No.	Swiss-Prot AC	Distance	Protein Name
1028	Q9Y5P8	0.2180	Serine/threonine-protein phosphatase 2A 48 kDa regulatory subunit B
1029	Q8IV36	0.2180	Uncharacterized protein C17orf28
1030	P63244	0.2178	Guanine nucleotide-binding protein subunit beta 2-like 1
1031	P28062	0.2175	Proteasome subunit beta type 8
1032	Q9BXT6	0.2160	Putative helicase Mov10l1
1033	P14902	0.2156	Indoleamine 2,3-dioxygenase
1034	Q8NCT1	0.2144	Arrestin domain-containing protein 4
1035	Q8IWG1	0.2142	WD repeat protein 63
1036	Q96MR6	0.2137	WD repeat protein 65
1037	P51810	0.2127	G-protein coupled receptor 143
1038	Q8NBE8	0.2114	Kelch-like protein 23
1039	Q8N865	0.2113	Uncharacterized protein C7orf31
1040	Q9NXW9	0.2081	Alkylated repair protein alkB homolog 4
1041	Q8NI36	0.2079	WD repeat protein 36
1042	Q8IXJ6	0.2076	NAD-dependent deacetylase sirtuin-2
1043	Q8TDN4	0.2032	CDK5 and ABL1 enzyme substrate 1
1044	P57740	0.2025	Nuclear pore complex protein Nup107
1045	Q9Y5U5	0.2001	Tumor necrosis factor receptor superfamily member 18
1046	Q8ND90	0.1984	Paraneoplastic antigen Ma1
1047	Q9Y5R4	0.1976	HemK methyltransferase family member 1
1048	Q8TC26	0.1975	Transmembrane protein 163
1049	O15397	0.1975	Importin-8
1050	Q9Y2T2	0.1963	AP-3 complex subunit mu-1
1051	P52961	0.1961	GPI-linked NAD(P)(+)--arginine ADP-ribosyltransferase 1
1052	Q9ULQ0	0.1960	Protein FAM40B
1053	O14531	0.1959	Dihydropyrimidinase-related protein 4
1054	Q9UNN5	0.1947	FAS-associated factor 1
1055	Q9P0I2	0.1944	Transmembrane protein 111
1056	Q6Q0C0	0.1939	E3 ubiquitin-protein ligase TRAF7
1057	Q96RT8	0.1922	Gamma-tubulin complex component 5
1058	Q9UQG0	0.1921	HERV-K_3q27.3 provirus ancestral Pol protein
1059	Q14397	0.1917	Glucokinase regulatory protein
1060	Q6DCA0	0.1909	AMMECR1-like protein
1061	Q5MNZ9	0.1908	WD repeat domain phosphoinositide-interacting protein 1
1062	Q9H269	0.1907	Vacuolar protein sorting-associated protein 16 homolog
1063	P04424	0.1906	Argininosuccinate lyase
1064	Q96A28	0.1904	SLAM family member 9
1065	P51606	0.1904	N-acylglucosamine 2-epimerase
1066	O43809	0.1902	Cleavage and polyadenylation specificity factor 5
1067	Q9BX95	0.1891	Sphingosine-1-phosphate phosphatase 1
1068	Q9BYJ9	0.1887	YTH domain family protein 1
1069	Q0ZLH3	0.1885	Pejvakin
1070	Q9Y2G5	0.1878	GDP-fucose protein O-fucosyltransferase 2

No.	Swiss-Prot AC	Distance	Protein Name
1071	Q8NEM8	0.1877	Cytosolic carboxypeptidase 3
1072	Q9H4D5	0.1874	Nuclear RNA export factor 3
1073	Q9H999	0.1869	Pantothenate kinase 3
1074	O60347	0.1868	TBC1 domain family member 12
1075	Q2T9K0	0.1864	Transmembrane protein 44
1076	Q68D06	0.1860	Schlafen family member 13
1077	Q13087	0.1845	Protein disulfide-isomerase A2
1078	P17643	0.1835	5,6-dihydroxyindole-2-carboxylic acid oxidase
1079	P54868	0.1833	Hydroxymethylglutaryl-CoA synthase, mitochondrial
1080	Q8N2U9	0.1824	PQ-loop repeat-containing protein 1
1081	O75691	0.1819	Small subunit processome component 20 homolog
1082	Q5VXU1	0.1817	T-cell lymphoma breakpoint associated target protein 1
1083	O43868	0.1811	Sodium/nucleoside cotransporter 2
1084	Q6NXR0	0.1810	Interferon-inducible GTPase 5
1085	Q9BXJ3	0.1798	Complement C1q tumor necrosis factor-related protein 4
1086	Q96MF6	0.1796	Protein COQ10 A, mitochondrial
1087	Q9H841	0.1792	NIPA-like protein 2
1088	Q15031	0.1783	Probable leucyl-tRNA synthetase, mitochondrial
1089	P78346	0.1775	Ribonuclease P protein subunit p30
1090	Q9UIJ5	0.1774	Palmitoyltransferase ZDHHC2
1091	Q8NHP8	0.1761	LAMA-like protein 2
1092	Q13867	0.1761	Bleomycin hydrolase
1093	Q9GZS0	0.1756	Dynein intermediate chain 2, axonemal
1094	O95807	0.1749	Transmembrane protein 50A
1095	Q9BX51	0.1741	Gamma-glutamyltransferase-like protein 6
1096	Q8TDY3	0.1741	Actin-related protein T2
1097	P49279	0.1739	Natural resistance-associated macrophage protein 1
1098	O75604	0.1739	Ubiquitin carboxyl-terminal hydrolase 2
1099	Q7Z4G4	0.1724	tRNA guanosine-2'-O-methyltransferase TRM11 homolog
1100	Q5TD97	0.1723	Four and a half LIM domains protein 5
1101	P60900	0.1721	Proteasome subunit alpha type 6
1102	O15160	0.1704	DNA-directed RNA polymerase I 40 kDa polypeptide
1103	Q49AN0	0.1703	Cryptochrome-2
1104	Q9H6U6	0.1700	Breast carcinoma amplified sequence 3
1105	O95445	0.1699	Apolipoprotein M
1106	Q12770	0.1694	Sterol regulatory element-binding protein cleavage-activating protein
1107	Q8WV07	0.1688	Oral cancer overexpressed protein 1
1108	O15503	0.1688	Insulin-induced gene 1 protein
1109	Q6ZRR5	0.1681	Transmembrane protein 136
1110	P0C2W1	0.1680	F-box/SPRY domain-containing protein 1
1111	Q14315	0.1675	Filamin-C
1112	Q96BW1	0.1662	Uracil phosphoribosyltransferase
1113	Q86W28	0.1655	NACHT, LRR and PYD domains-containing protein 8

No.	Swiss-Prot AC	Distance	Protein Name
1114	Q9Y2P5	0.1650	Bile acyl-CoA synthetase
1115	Q6NW40	0.1618	RGM domain family member B
1116	Q7Z739	0.1613	YTH domain family protein 3
1117	P60201	0.1609	Myelin proteolipid protein
1118	Q6ZMZ0	0.1598	IBR domain-containing protein 3
1119	Q14353	0.1594	Guanidinoacetate N-methyltransferase
1120	Q9HA72	0.1591	Protein FAM26B
1121	O14746	0.1586	Telomerase reverse transcriptase
1122	Q3MJ13	0.1585	WD repeat protein 72
1123	Q9HBV2	0.1577	Sperm acrosome membrane-associated protein 1
1124	Q9P2N7	0.1568	Kelch-like protein 13
1125	Q96CW1	0.1564	AP-2 complex subunit mu-1
1126	Q9H7F4	0.1539	Transmembrane protein 185B
1127	P33991	0.1538	DNA replication licensing factor MCM4
1128	O75083	0.1536	WD repeat protein 1
1129	Q9Y217	0.1521	Myotubularin-related protein 6
1130	Q96I51	0.1516	Williams-Beuren syndrome chromosome region 16 protein
1131	Q04671	0.1515	P protein
1132	Q9H4L4	0.1506	Sentrin-specific protease 3
1133	O60522	0.1479	Tudor domain-containing protein 6
1134	Q9NXR7	0.1469	Protein BRE
1135	O75426	0.1464	F-box only protein 24
1136	Q96SL4	0.1452	Glutathione peroxidase 7
1137	P06133	0.1446	UDP-glucuronosyltransferase 2B4
1138	Q9NRG9	0.1444	Aladin
1139	Q13530	0.1441	Serine incorporator 3
1140	Q96JB1	0.1437	Ciliary dynein heavy chain 8
1141	P53992	0.1429	Protein transport protein Sec24C
1142	A0FGR8	0.1428	Protein FAM62B
1143	Q8NEG0	0.1425	Protein FAM71C
1144	Q9Y6U3	0.1424	Adseverin
1145	Q3B7J2	0.1401	Glucose-fructose oxidoreductase domain-containing protein 2
1146	Q13564	0.1394	NEDD8-activating enzyme E1 regulatory subunit
1147	Q9Y2G8	0.1388	DnaJ homolog subfamily C member 16
1148	Q12765	0.1373	Secernin-1
1149	Q5JW98	0.1360	Protein FAM26D
1150	Q9UKW6	0.1357	ETS-related transcription factor Elf-5
1151	O96006	0.1345	Zinc finger BED domain-containing protein 1
1152	Q92993	0.1321	Histone acetyltransferase HTATIP
1153	P04843	0.1318	Dolichyl-diphosphooligosaccharide--protein glycosyltransferase 67 kDa subunit
1154	Q96EW2	0.1315	HSPB1-associated protein 1
1155	Q6N075	0.1310	Major facilitator superfamily domain-containing protein 5
1156	Q9HC58	0.1304	Sodium/potassium/calcium exchanger 3

No.	Swiss-Prot AC	Distance	Protein Name
1157	Q8TAT6	0.1294	Nuclear protein localization protein 4 homolog
1158	Q96KG9	0.1290	N-terminal kinase-like protein
1159	Q9BT30	0.1271	Alkylated repair protein alkB homolog 7
1160	Q8WXE9	0.1261	Stonin-2
1161	Q96JJ3	0.1259	Engulfment and cell motility protein 2
1162	Q9UH17	0.1249	Probable DNA dC-
1163	Q9NWX6	0.1247	Probable tRNA(His) guanylyltransferase
1164	Q9BZ71	0.1243	Membrane-associated phosphatidylinositol transfer protein 3
1165	O00526	0.1236	Uroplakin-2
1166	Q9HBU6	0.1227	Ethanolamine kinase 1
1167	Q9UJX0	0.1202	Oxidative stress induced growth inhibitor 1
1168	P54922	0.1193	[Protein ADP-ribosylarginine] hydrolase
1169	Q8TCF1	0.1187	AN1-type zinc finger protein 1
1170	Q14195	0.1181	Dihydropyrimidinase-related protein 3
1171	Q96MK3	0.1178	Protein FAM20A
1172	Q14997	0.1178	Proteasome activator complex subunit 4
1173	Q8IV42	0.1172	L-seryl-tRNA(Sec) kinase
1174	Q96BY7	0.1169	Uncharacterized protein C14orf103
1175	P82675	0.1167	Mitochondrial 28S ribosomal protein S5
1176	Q9HCE1	0.1164	Putative helicase MOV-10
1177	P19404	0.1153	NADH dehydrogenase [ubiquinone] flavoprotein 2, mitochondrial
1178	Q9Y2Q3	0.1146	Glutathione S-transferase kappa 1
1179	Q9Y277	0.1141	Voltage-dependent anion-selective channel protein 3
1180	Q96RL7	0.1132	Vacuolar protein sorting-associated protein 13A
1181	Q86XE5	0.1132	Dihydrodipicolinate synthase-like, mitochondrial
1182	Q9Y223	0.1131	Bifunctional UDP-N-acetylglucosamine 2-epimerase/N-acetylmannosamine kinase
1183	Q8TCT0	0.1131	Ceramide kinase
1184	P55265	0.1126	Double-stranded RNA-specific adenosine deaminase
1185	Q8TE73	0.1122	Ciliary dynein heavy chain 5
1186	Q9NYU1	0.1118	UDP-glucose:glycoprotein glucosyltransferase 2
1187	P29120	0.1116	Neuroendocrine convertase 1
1188	Q9UJA2	0.1108	Cardiolipin synthetase
1189	Q14019	0.1103	Coactosin-like protein
1190	Q8NFP9	0.1095	Protein neurobeachin
1191	O43511	0.1095	Pendrin
1192	O00418	0.1080	Elongation factor 2 kinase
1193	Q5T4F7	0.1062	Secreted frizzled-related protein 5
1194	Q9Y2A7	0.1056	Nck-associated protein 1
1195	Q9HDD0	0.1035	HRAS-like suppressor
1196	Q8TCS8	0.1034	Polyribonucleotide nucleotidyltransferase 1, mitochondrial
1197	Q9UBL9	0.1026	P2X purinoceptor 2
1198	Q9NST1	0.1010	Adiponutrin
1199	Q969V3	0.1006	Nicalin

No.	Swiss-Prot AC	Distance	Protein Name
1200	Q9BR09	0.0993	Neuralized-like protein 2
1201	Q96K19	0.0992	RING finger protein 170
1202	Q9HDB5	0.0991	Neurexin-3-beta
1203	Q6ZQW0	0.0988	Indoleamine 2,3-dioxygenase-like protein 1
1204	Q7Z7G8	0.0987	Vacuolar protein sorting-associated protein 13B
1205	Q99755	0.0985	Phosphatidylinositol-4-phosphate 5-kinase type-1 alpha
1206	Q9UJ55	0.0979	MAGE-like protein 2
1207	O60716	0.0976	Catenin delta-1
1208	Q9Y5Q8	0.0969	General transcription factor 3C polypeptide 5
1209	O75419	0.0958	CDC45-related protein
1210	Q8NCL8	0.0957	Transmembrane protein 116
1211	Q7Z5M5	0.0957	Transmembrane channel-like protein 3
1212	Q9HB90	0.0955	Ras-related GTP-binding protein C
1213	Q14145	0.0948	Kelch-like ECH-associated protein 1
1214	Q16555	0.0928	Dihydropyrimidinase-related protein 2
1215	P50461	0.0924	Cysteine and glycine-rich protein 3
1216	Q8N9N2	0.0923	Activating signal cointegrator 1 complex subunit 1
1217	Q13724	0.0922	Mannosyl-oligosaccharide glucosidase
1218	Q8N966	0.0907	Putative palmitoyltransferase ZDHHC22
1219	O15457	0.0884	MutS protein homolog 4
1220	Q9Y2Z2	0.0881	Protein MTO1 homolog, mitochondrial
1221	Q9BSY9	0.0881	UPF0326 protein C1orf121
1222	Q96EB1	0.0881	Elongator complex protein 4
1223	Q08334	0.0878	Interleukin-10 receptor beta chain
1224	Q9H0E7	0.0870	Ubiquitin carboxyl-terminal hydrolase 44
1225	Q6Y288	0.0865	Beta-1,3-glucosyltransferase
1226	O60476	0.0846	Mannosyl-oligosaccharide 1,2-alpha-mannosidase IB
1227	O75366	0.0840	Advillin
1228	P40937	0.0839	Replication factor C subunit 5
1229	Q15904	0.0838	Vacuolar ATP synthase subunit S1
1230	Q9UKT8	0.0835	F-box/WD repeat protein 2
1231	P27105	0.0833	Erythrocyte band 7 integral membrane protein
1232	P49736	0.0831	DNA replication licensing factor MCM2
1233	Q9NTN3	0.0829	UDP-glucuronic acid/UDP-N-acetylgalactosamine transporter
1234	Q9Y4I5	0.0827	Tesmin
1235	Q9P2C4	0.0817	Transmembrane protein 181
1236	Q9H1Y0	0.0802	Autophagy protein 5
1237	Q8NHH1	0.0793	Tubulin--tyrosine ligase-like protein 11
1238	Q9NSD9	0.0791	Phenylalanyl-tRNA synthetase beta chain
1239	Q8NFM7	0.0790	Interleukin-17 receptor D
1240	Q13614	0.0783	Myotubularin-related protein 2
1241	O43913	0.0772	Origin recognition complex subunit 5
1242	Q96FG2	0.0771	ELMO domain-containing protein 3

No.	Swiss-Prot AC	Distance	Protein Name
1243	Q9Y2M2	0.0764	Uncharacterized protein C3orf32
1244	P09958	0.0747	Furin
1245	Q15629	0.0743	Translocation-associated membrane protein 1
1246	Q9UL17	0.0737	T-box transcription factor TBX21
1247	Q6ZRP7	0.0735	Sulfhydryl oxidase 2
1248	P51784	0.0723	Ubiquitin carboxyl-terminal hydrolase 11
1249	P67812	0.0715	Signal peptidase complex catalytic subunit SEC11A
1250	O60729	0.0703	Dual specificity protein phosphatase CDC14B
1251	Q8N0S6	0.0701	Centromere protein L
1252	O00423	0.0700	Echinoderm microtubule-associated protein-like 1
1253	Q9UJH8	0.0699	Meteorin
1254	Q96MV8	0.0677	Palmitoyltransferase ZDHHC15
1255	Q969F9	0.0676	Hermansky-Pudlak syndrome 3 protein
1256	Q9NUT2	0.0674	ATP-binding cassette sub-family B member 8, mitochondrial
1257	Q8IUX4	0.0673	DNA dC-
1258	P61077	0.0673	Ubiquitin-conjugating enzyme E2 D3
1259	Q9NVQ4	0.0662	Fas apoptotic inhibitory molecule 1
1260	O15195	0.0656	Villin-like protein
1261	Q8TBX8	0.0647	Phosphatidylinositol-4-phosphate 5-kinase type-2 gamma
1262	Q07507	0.0624	Dermatopontin
1263	Q96JB6	0.0617	Lysyl oxidase homolog 4
1264	Q9UJT9	0.0614	F-box/LRR-repeat protein 7
1265	P14735	0.0614	Insulin-degrading enzyme
1266	P11047	0.0606	Laminin subunit gamma-1
1267	Q9Y5L3	0.0601	Ectonucleoside triphosphate diphosphohydrolase 2
1268	Q14032	0.0586	Bile acid CoA:amino acid N-acyltransferase
1269	Q53HC5	0.0582	Kelch-like protein 26
1270	Q9H3H5	0.0569	UDP-N-acetylglucosamine--dolichyl-phosphate N
1271	Q16526	0.0569	Cryptochrome-1
1272	Q92826	0.0568	Homeobox protein Hox-B13
1273	Q11206	0.0567	CMP-N-acetylneuraminate-beta-galactosamide-alpha-2,3-sialyltransferase
1274	Q5U5R9	0.0562	Probable E3 ubiquitin-protein ligase HECTD2
1275	P82933	0.0558	28S ribosomal protein S9, mitochondrial
1276	O14926	0.0553	Fascin-2
1277	Q9NV64	0.0552	Transmembrane protein 39A
1278	Q9NRR6	0.0527	72 kDa inositol polyphosphate 5-phosphatase
1279	Q14DG7	0.0524	Transmembrane protein 132B
1280	P45381	0.0520	Aspartoacylase
1281	Q10469	0.0516	Alpha-1,6-mannosyl-glycoprotein 2-beta-N-acetylglucosaminyltransferase
1282	Q5JUQ0	0.0507	Protein FAM78A
1283	P58401	0.0505	Neurexin-2-beta
1284	Q9UHJ6	0.0496	Carbohydrate kinase-like protein
1285	Q9NTM9	0.0495	Copper homeostasis protein cutC homolog

No.	Swiss-Prot AC	Distance	Protein Name
1286	P41247	0.0492	Patatin-like phospholipase domain-containing protein 4
1287	Q96JH7	0.0491	Deubiquitinating protein VCIP135
1288	Q7Z419	0.0491	E3 ubiquitin-protein ligase IBRDC2
1289	Q96F86	0.0489	Enhancer of mRNA-decapping protein 3
1290	O60318	0.0485	80 kDa MCM3-associated protein
1291	Q7L5Y6	0.0482	DET1 homolog
1292	Q15063	0.0475	Periostin
1293	Q96CP7	0.0466	TLC domain-containing protein 1
1294	Q6X4W1	0.0448	Nasal embryonic luteinizing hormone-releasing hormone factor
1295	P35125	0.0437	Ubiquitin carboxyl-terminal hydrolase 6
1296	Q9Y4L5	0.0436	Zinc finger protein 364
1297	Q96T60	0.0431	Bifunctional polynucleotide phosphatase/kinase
1298	Q9H2T7	0.0423	Ran-binding protein 17
1299	Q969S0	0.0423	UDP-xylose and UDP-N-acetylglucosamine transporter
1300	O75132	0.0419	Zinc finger BED domain-containing protein 4
1301	Q75V66	0.0416	Transmembrane protein 16E
1302	O60930	0.0415	Ribonuclease H1
1303	P49257	0.0398	ERGIC-53 protein
1304	P78333	0.0393	Glypican-5
1305	Q99436	0.0386	Proteasome subunit beta type 7
1306	Q9H981	0.0383	Actin-related protein 8
1307	P62837	0.0382	Ubiquitin-conjugating enzyme E2 D2
1308	Q8WWR8	0.0381	Sialidase-4
1309	P46459	0.0376	Vesicle-fusing ATPase
1310	P29017	0.0374	T-cell surface glycoprotein CD1c
1311	Q13939	0.0369	Calicin
1312	O95759	0.0361	TBC1 domain family member 8
1313	O95278	0.0355	Laforin
1314	Q9Y2X0	0.0352	Thyroid hormone receptor-associated protein complex 95 kDa component
1315	O75845	0.0343	Lathosterol oxidase
1316	Q9UKF5	0.0342	ADAM 29
1317	Q6ZRI8	0.0315	Putative Rho GTPase-activating protein FLJ46335
1318	Q8NFB2	0.0306	Transmembrane protein 185A
1319	P42785	0.0304	Lysosomal Pro-X carboxypeptidase
1320	Q6ZVK1	0.0294	Transmembrane protein 179
1321	P57071	0.0271	PR domain zinc finger protein 15
1322	P11279	0.0267	Lysosome-associated membrane glycoprotein 1
1323	P36269	0.0263	Gamma-glutamyltransferase 5
1324	Q8N1G2	0.0262	Protein KIAA0082
1325	Q9Y2L5	0.0260	Protein TRS85 homolog
1326	O60725	0.0260	Protein-S-isoprenylcysteine O-methyltransferase
1327	P48664	0.0251	Excitatory amino acid transporter 4
1328	Q96AX1	0.0250	Vacuolar protein sorting-associated protein 33A

No.	Swiss-Prot AC	Distance	Protein Name
1329	Q7KZF4	0.0240	Staphylococcal nuclease domain-containing protein 1
1330	Q6YHK3	0.0235	CD109 antigen
1331	Q92901	0.0228	60S ribosomal protein L3-like
1332	O75829	0.0224	Chondromodulin-1
1333	Q8TC59	0.0212	Piwi-like protein 2
1334	Q9Y216	0.0205	Myotubularin-related protein 7
1335	Q6ZRQ5	0.0205	Uncharacterized protein C6orf167
1336	Q9NQC7	0.0195	Probable ubiquitin carboxyl-terminal hydrolase CYLD
1337	Q2TAY7	0.0189	Smu-1 suppressor of mec-8 and unc-52 protein homolog
1338	O00337	0.0186	Sodium/nucleoside cotransporter 1
1339	O00755	0.0183	Protein Wnt-7a
1340	Q9H4H8	0.0174	Protein FAM83D
1341	P47985	0.0160	Ubiquinol-cytochrome c reductase iron-sulfur subunit, mitochondrial
1342	Q86XP6	0.0153	Gastrokine-2
1343	O75695	0.0149	Protein XRP2
1344	Q9H0V9	0.0144	VIP36-like protein
1345	Q96CN7	0.0143	Isochorismatase domain-containing protein 1
1346	Q5JWF2	0.0143	Guanine nucleotide-binding protein G(s) subunit alpha isoforms XLas
1347	O43427	0.0141	Acidic fibroblast growth factor intracellular-binding protein
1348	P61647	0.0136	Alpha-2,8-sialyltransferase 8F
1349	Q7Z7B1	0.0135	Phosphatidylinositol-glycan biosynthesis class W protein
1350	P57678	0.0133	Component of gems 4
1351	Q9HC96	0.0128	Calpain-10
1352	Q86U70	0.0121	LIM domain-binding protein 1
1353	O95674	0.0112	Phosphatidate cytidylyltransferase 2
1354	Q13145	0.0107	BMP and activin membrane-bound inhibitor homolog
1355	Q9GZU3	0.0101	Transmembrane protein 39B
1356	Q7L576	0.0101	Cytoplasmic FMR1-interacting protein 1
1357	Q9BZQ4	0.0098	Nicotinamide mononucleotide adenylyltransferase 2
1358	Q9P2M4	0.0095	TBC1 domain family member 14
1359	Q16531	0.0083	DNA damage-binding protein 1
1360	Q9UKV8	0.0079	Eukaryotic translation initiation factor 2C 2
1361	P16278	0.0078	Beta-galactosidase
1362	O95147	0.0077	Dual specificity protein phosphatase 14
1363	P63132	0.0073	HERV-K_19p13.11 provirus ancestral Pol protein
1364	Q8NEG7	0.0068	Protein FAM116B
1365	Q9HBV1	0.0066	Popeye domain-containing protein 3
1366	Q15181	0.0063	Inorganic pyrophosphatase
1367	O14791	0.0063	Apolipoprotein-L1
1368	Q5VSL9	0.0061	Protein FAM40A
1369	Q8TAP6	0.0056	Centrosomal protein of 76 kDa
1370	Q14139	0.0056	Ubiquitin conjugation factor E4 A
1371	P47897	0.0054	Glutaminyl-tRNA synthetase

No.	Swiss-Prot AC	Distance	Protein Name
1372	Q8IU89	0.0043	LAG1 longevity assurance homolog 3
1373	Q9NP92	0.0042	Mitochondrial 28S ribosomal protein S30
1374	Q9NYW6	0.0035	Taste receptor type 2 member 3
1375	O95602	0.0033	DNA-directed RNA polymerase I subunit RPA1
1376	Q9Y2B2	0.0026	N-acetylglucosaminyl-phosphatidylinositol de-N-acetylase
1377	Q8WVM8	0.0026	Sec1 family domain-containing protein 1
1378	P07942	0.0017	Laminin subunit beta-1
1379	Q86VN1	0.0012	Vacuolar protein sorting-associated protein 36
1380	P06396	0.0010	Gelsolin
1381	Q9UPQ8	0.0005	Transmembrane protein 15
1382	Q70CQ2	0.0005	Ubiquitin carboxyl-terminal hydrolase 34
1383	P62879	0.0004	Guanine nucleotide-binding protein G(I)/G(S)/G(T) subunit beta 2
