# Supplementary material for: Prediction of potential drug targets based on simple sequence properties
Source: BMC Bioinformatics. 2007 Sep 20;8:353. doi: 10.1186/1471-2105-8-353 (PMC2082046; doi:10.1186/1471-2105-8-353)
Supplement: Additional file 2 — 186 targets of FDA-approved oral small-molecular drugs. Total 186 targets of FDA-approved oral small-molecular drugs. The SwissProt ID and protein name was in it. [file 1471-2105-8-353-S2.pdf]

Table S2. 186 targets of FDA-approved oral small-molecular drugs

No.	Swiss-Prot AC	Protein Name
1	O14732	Inositol monophosphatase 2
2	O14920	Inhibitor of nuclear factor kappa-B kinase subunit beta
3	O43451	Maltase-glucoamylase, intestinal [Includes: Maltase
4	O60706	ATP-binding cassette transporter sub-family C member 9
5	O76074	cGMP-specific 3',5'-cyclic phosphodiesterase
6	O95180	Voltage-dependent T-type calcium channel subunit alpha-1H
7	P00352	Retinal dehydrogenase 1
8	P00374	Dihydrofolate reductase
9	P00519	Proto-oncogene tyrosine-protein kinase ABL1
10	P00533	Epidermal growth factor receptor
11	P00747	Plasminogen
12	P00750	Tissue-type plasminogen activator
13	P00813	Adenosine deaminase
14	P00918	Carbonic anhydrase 2
15	P03372	Estrogen receptor
16	P04035	3-hydroxy-3-methylglutaryl-coenzyme A reductase
17	P04049	RAF proto-oncogene serine/threonine-protein kinase
18	P04150	Glucocorticoid receptor
19	P04746	Pancreatic alpha-amylase
20	P04818	Thymidylate synthase
21	P05023	Sodium/potassium-transporting ATPase alpha-1 chain
22	P05108	Cytochrome P450 11A1, mitochondrial
23	P05184	Cytochrome P450 3A3
24	P06239	Proto-oncogene tyrosine-protein kinase LCK
25	P06241	Proto-oncogene tyrosine-protein kinase Fyn
26	P06276	Cholinesterase
27	P06401	Progesterone receptor
28	P06858	Lipoprotein lipase
29	P07098	Gastric triacylglycerol lipase
30	P07099	Epoxide hydrolase 1
31	P07101	Tyrosine 3-monooxygenase
32	P07202	Thyroid peroxidase
33	P07437	Tubulin beta chain
34	P07550	Beta-2 adrenergic receptor
35	P07947	Proto-oncogene tyrosine-protein kinase Yes
36	P07949	Proto-oncogene tyrosine-protein kinase receptor ret
37	P08172	Muscarinic acetylcholine receptor M2
38	P08173	Muscarinic acetylcholine receptor M4

No.	Swiss-Prot AC	Protein Name
39	P08235	Mineralocorticoid receptor
40	P08588	Beta-1 adrenergic receptor
41	P08908	5-hydroxytryptamine 1A receptor
42	P08912	Muscarinic acetylcholine receptor M5
43	P08913	Alpha-2A adrenergic receptor
44	P09619	Beta platelet-derived growth factor receptor
45	P09917	Arachidonate 5-lipoxygenase
46	P10275	Androgen receptor
47	P10276	Retinoic acid receptor alpha
48	P10721	Mast/stem cell growth factor receptor
49	P10826	Retinoic acid receptor beta
50	P10827	Thyroid hormone receptor alpha
51	P10828	Thyroid hormone receptor beta-1
52	P11229	Muscarinic acetylcholine receptor M1
53	P11388	DNA topoisomerase 2-alpha
54	P11473	Vitamin D3 receptor
55	P11511	Cytochrome P450 19A1
56	P12235	ADP/ATP translocase 1
57	P12268	Inosine-5'-monophosphate dehydrogenase 2
58	P12821	Angiotensin-converting enzyme, somatic isoform
59	P12931	Proto-oncogene tyrosine-protein kinase Src
60	P13631	Retinoic acid receptor gamma-1
61	P14060	3 beta-hydroxysteroid dehydrogenase/Delta 5--4-isomerase type I
62	P14324	Farnesyl pyrophosphate synthetase
63	P14416	D(2) dopamine receptor
64	P14867	Gamma-aminobutyric-acid receptor subunit alpha-1
65	P15056	B-Raf proto-oncogene serine/threonine-protein kinase
66	P15085	Carboxypeptidase A1
67	P15121	Aldose reductase
68	P15509	Granulocyte-macrophage colony-stimulating factor receptor alpha chain
69	P15538	Cytochrome P450 11B1, mitochondrial
70	P16233	Pancreatic triacylglycerol lipase
71	P16234	Alpha platelet-derived growth factor receptor
72	P17787	Neuronal acetylcholine receptor subunit beta-2
73	P17948	Vascular endothelial growth factor receptor 1
74	P18089	Alpha-2B adrenergic receptor
75	P18405	3-oxo-5-alpha-steroid 4-dehydrogenase 1
76	P18507	Gamma-aminobutyric-acid receptor subunit gamma-2
77	P18825	Alpha-2C adrenergic receptor
78	P20309	Muscarinic acetylcholine receptor M3
79	P20648	Potassium-transporting ATPase alpha chain 1

No.	Swiss-Prot AC	Protein Name
80	P20711	Aromatic-L-amino-acid decarboxylase
81	P20839	Inosine-5'-monophosphate dehydrogenase 1
82	P21397	Amine oxidase [flavin-containing] A
83	P21554	Cannabinoid receptor 1
84	P21728	D(1A) dopamine receptor
85	P21817	Ryanodine receptor 1
86	P21917	D(4) dopamine receptor
87	P21918	D(1B) dopamine receptor
88	P21964	Catechol O-methyltransferase
89	P22303	Acetylcholinesterase
90	P23219	Prostaglandin G/H synthase 1
91	P23284	Peptidyl-prolyl cis-trans isomerase B
92	P23975	Sodium-dependent noradrenaline transporter
93	P24530	Endothelin B receptor
94	P25021	Histamine H2 receptor
95	P25100	Alpha-1D adrenergic receptor
96	P25101	Endothelin-1 receptor
97	P25103	Substance-P receptor
98	P27338	Amine oxidase [flavin-containing] B
99	P27815	cAMP-specific 3',5'-cyclic phosphodiesterase 4A
100	P28221	5-hydroxytryptamine 1D receptor
101	P28222	5-hydroxytryptamine 1B receptor
102	P28223	5-hydroxytryptamine 2A receptor
103	P28335	5-hydroxytryptamine 2C receptor
104	P29274	Adenosine A2a receptor
105	P29275	Adenosine A2b receptor
106	P29317	Ephrin type-A receptor 2
107	P30518	Vasopressin V2 receptor
108	P30531	Sodium- and chloride-dependent GABA transporter 1
109	P30542	Adenosine A1 receptor
110	P30556	Type-1 angiotensin II receptor
111	P30939	5-hydroxytryptamine 1F receptor
112	P31213	3-oxo-5-alpha-steroid 4-dehydrogenase 2
113	P31350	Ribonucleoside-diphosphate reductase M2 subunit
114	P31645	Sodium-dependent serotonin transporter
115	P32754	4-hydroxyphenylpyruvate dioxygenase
116	P33765	Adenosine A3 receptor
117	P34972	Cannabinoid receptor 2
118	P35348	Alpha-1A adrenergic receptor
119	P35354	Prostaglandin G/H synthase 2
120	P35367	Histamine H1 receptor

No.	Swiss-Prot AC	Protein Name
121	P35368	Alpha-1B adrenergic receptor
122	P35372	Mu-type opioid receptor
123	P35462	D(3) dopamine receptor
124	P35498	Sodium channel protein type 1 subunit alpha
125	P35499	Sodium channel protein type 4 subunit alpha
126	P35916	Vascular endothelial growth factor receptor 3
127	P35968	Vascular endothelial growth factor receptor 2
128	P36888	FL cytokine receptor
129	P37088	Amiloride-sensitive sodium channel subunit alpha
130	P37231	Peroxisome proliferator-activated receptor gamma
131	P37288	Vasopressin V1a receptor
132	P38606	Vacuolar ATP synthase catalytic subunit A
133	P39086	Glutamate receptor, ionotropic kainate 1
134	P41143	Delta-type opioid receptor
135	P41145	Kappa-type opioid receptor
136	P41146	Nociceptin receptor
137	P41180	Extracellular calcium-sensing receptor
138	P41595	5-hydroxytryptamine 2B receptor
139	P42261	Glutamate receptor 1
140	P43116	Prostaglandin E2 receptor EP2 subtype
141	P43681	Neuronal acetylcholine receptor subunit alpha-4
142	P46098	5-hydroxytryptamine 3 receptor
143	P47870	Gamma-aminobutyric-acid receptor subunit beta-2
144	P47901	Vasopressin V1b receptor
145	P47989	Xanthine dehydrogenase/oxidase
146	P48039	Melatonin receptor type 1A
147	P49286	Melatonin receptor type 1B
148	P49895	Type I iodothyronine deiodinase
149	P51649	Succinate semialdehyde dehydrogenase, mitochondrial
150	P51787	Potassium voltage-gated channel subfamily KQT member 1
151	P54289	Dihydropyridine-sensitive L-type calcium channel subunits alpha- 2/delta
152	P55017	Solute carrier family 12 member 3
153	P62937	Peptidyl-prolyl cis-trans isomerase A
154	P62942	FK506-binding protein 1A
155	P63316	Troponin C, slow skeletal and cardiac muscles
156	P80404	4-aminobutyrate aminotransferase, mitochondrial
157	Q01959	Sodium-dependent dopamine transporter
158	Q02127	Dihydroorotate dehydrogenase, mitochondrial
159	Q02153	Guanylate cyclase soluble subunit beta-1
160	Q05940	Synaptic vesicular amine transporter
161	Q07869	Peroxisome proliferator-activated receptor alpha

No.	Swiss-Prot AC	Protein Name
162	Q09428	ATP-binding cassette transporter sub-family C member 8
163	Q12809	Potassium voltage-gated channel subfamily H member 2
164	Q12879	Glutamate [NMDA] receptor subunit epsilon-1
165	Q13224	Glutamate [NMDA] receptor subunit epsilon-2
166	Q13621	Solute carrier family 12 member 1
167	Q13639	5-hydroxytryptamine 4 receptor
168	Q13936	Voltage-dependent L-type calcium channel subunit alpha-1C
169	Q14432	cGMP-inhibited 3',5'-cyclic phosphodiesterase A
170	Q14524	Sodium channel protein type 5 subunit alpha
171	Q14654	ATP-sensitive inward rectifier potassium channel 11
172	Q15413	Ryanodine receptor 3
173	Q15822	Neuronal acetylcholine receptor subunit alpha-2
174	Q16739	Ceramide glucosyltransferase
175	Q16881	Thioredoxin reductase 1, cytoplasmic
176	Q92731	Estrogen receptor beta
177	Q96RI1	Bile acid receptor
178	Q99720	Sigma 1-type opioid receptor
179	Q99808	Equilibrative nucleoside transporter 1
180	Q9BQB6	Vitamin K epoxide reductase complex subunit 1
181	Q9H244	P2Y purinoceptor 12
182	Q9H4B7	Tubulin beta-1 chain
183	Q9UBS5	Gamma-aminobutyric acid type B receptor, subunit 1
184	Q9UHC9	Niemann-Pick C1-like protein 1
185	Q9UK17	Potassium voltage-gated channel subfamily D member 3
186	Q9Y271	Cysteinyl leukotriene receptor 1
